# A practical guide for combining functional regions of interest and white matter bundles

**DOI:** 10.3389/fnins.2024.1385847

**Published:** 2024-08-16

**Authors:** Steven L. Meisler, Emily Kubota, Mareike Grotheer, John D. E. Gabrieli, Kalanit Grill-Spector

**Affiliations:** ^1^Program in Speech and Hearing Bioscience and Technology, Harvard Medical School, Boston, MA, United States; ^2^Department of Brain and Cognitive Sciences, Massachusetts Institute of Technology, Cambridge, MA, United States; ^3^Department of Psychology, Stanford University, Stanford, CA, United States; ^4^Department of Psychology, Philipps-Universität Marburg, Marburg, Germany; ^5^Center for Mind, Brain and Behavior – CMBB, Philipps-Universität Marburg and Justus-Liebig-Universität Giessen, Marburg, Germany; ^6^McGovern Institute for Brain Research, Massachusetts Institute of Technology, Cambridge, MA, United States; ^7^Wu Tsai Neurosciences Institute, Stanford University, Stanford, CA, United States

**Keywords:** DWI, fMRI, white matter, structural connectivity, open-source software

## Abstract

Diffusion-weighted imaging (DWI) is the primary method to investigate macro- and microstructure of neural white matter *in vivo*. DWI can be used to identify and characterize individual-specific white matter bundles, enabling precise analyses on hypothesis-driven connections in the brain and bridging the relationships between brain structure, function, and behavior. However, cortical endpoints of bundles may span larger areas than what a researcher is interested in, challenging presumptions that bundles are specifically tied to certain brain functions. Functional MRI (fMRI) can be integrated to further refine bundles such that they are restricted to functionally-defined cortical regions. Analyzing properties of these Functional Sub-Bundles (FSuB) increases precision and interpretability of results when studying neural connections supporting specific tasks. Several parameters of DWI and fMRI analyses, ranging from data acquisition to processing, can impact the efficacy of integrating functional and diffusion MRI. Here, we discuss the applications of the FSuB approach, suggest best practices for acquiring and processing neuroimaging data towards this end, and introduce the *FSuB-Extractor*, a flexible open-source software for creating FSuBs. We demonstrate our processing code and the *FSuB-Extractor* on an openly-available dataset, the Natural Scenes Dataset.

## 1 Introduction

White matter pathways are essential for communication among brain regions that orchestrate perception, cognition, and action (Fields, 2008; Filley and Fields, 2016). Functional relevance of white matter has been established since Carl Wernicke’s descriptions of aphasia in 1874 (Wernicke, 1874), in which lesions of the arcuate fasciculus led to impairments in speech production due to severed communication between inferior frontal and superior temporal regions. Beyond such clinical cases, often termed “disconnection syndromes” (Geschwind, 1965), variations in white matter microstructure are also reflected in individual differences in typical cognitive functions (Johansen-Berg, 2010; Roberts et al., 2013). Plasticity in white matter, which is central to learning, memory, and development (Sampaio-Baptista and Johansen-Berg, 2017; Fandakova and Hartley, 2020; Xin and Chan, 2020), is thought to be regulated by neural activity (Fields, 2015; de Faria et al., 2021). This collectively suggests there is a dynamic and causal interplay between white matter structure and brain function underlying typical and clinical cognition.

Diffusion-weighted imaging (DWI) is the primary method for investigating white matter *in vivo* (Basser et al., 1994), and can be used to infer structural properties of white matter more nuanced than gross volumetric estimates derived from standard anatomical imaging. White matter is organized into short-range association fibers and long-range bundles or tracts (Bullock et al., 2022). Conventional DWI can be used to resolve long-range bundles based on the strength and directionality of the underlying diffusion-weighted signal, often coupled with bundle-specific criteria such as atlas-based inclusion/exclusion areas or model-based clustering (Zhang et al., 2022). When done properly, bundle reconstruction is reliable and corresponds well with ground-truth white matter dissection (Schilling et al., 2020) and simulated phantom connectivity (Girard et al., 2023). These bundles connect gray matter regions and hence form the structural connectivity foundation for large-scale distributed neural networks that are associated with many cognitive functions (Bressler and Menon, 2010; Wang et al., 2015). By running analyses on the bundle-level instead of the voxel-level, investigators can examine specific connections in participant’s native brain space to precisely study the role of specific connections in the brain, mitigating concerns of multiple-comparisons across voxels and neuroanatomical dissimilarities across participants that often compromise whole-brain analyses (Van Hecke and Emsell, 2016). Such bundle-level analyses are implemented in various different software packages—such as AFQ (Yeatman et al., 2012; Kruper et al., 2021), TRACULA (Yendiki et al., 2011; Maffei et al., 2021), and the BUAN framework (Chandio et al., 2020)—and have been used in numerous studies.

**Figure 1:**
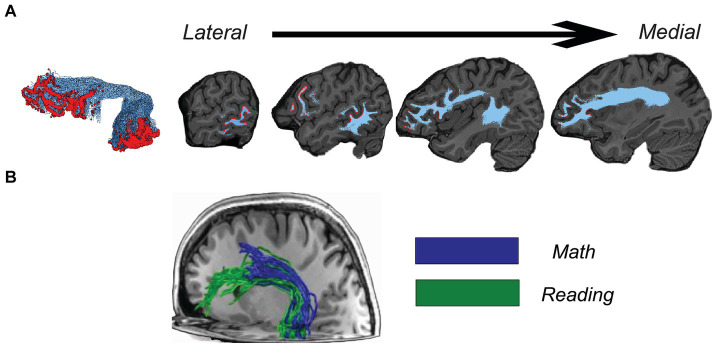
**(A)** Cortical endpoints (red) of the left arcuate fasciculus (light blue), overlaid on a T1-weighted image. Data come from the first subject of the Natural Scenes Dataset. **(B)** Functional sub-bundles of the left arcuate fasciculus that are specific to math (blue) and reading (green) in a representative adult. Adapted with permission from Grotheer et al. (2019) (under the Creative Commons Attribution 4.0 International License: http://creativecommons.org/licenses/by/4.0/).

However, an outstanding concern for linking white matter bundle properties to neural function or behavior is that the areas of cortex that bundles connect to are often larger than the fine-grained functional organization of the brain. As such, only a small sub-component of each bundle may be associated with the specific function or behavior being studied. For example, the superior longitudinal fasciculus is composed of several sub-bundles (Schurr et al., 2020). Similarly, separate sub-bundles within the arcuate fasciculus connect regions in the brain supporting reading and math, and, moreover, these sub-bundles have distinct microstructural profiles (Grotheer et al., 2019) (Figure 1). That these sub-bundles exist not only provides valuable insight into functional neuroanatomy, but also introduces an important methodological consideration, in that properties of these sub-bundles may serve as more theoretically-motivated metrics for gauging brain-behavior relationships. To resolve Functional Sub-Bundles (FSuB), one can integrate functional MRI (fMRI) in the bundle extraction process, identifying only those streamlines of a bundle that connect to the task-relevant functional regions of interest. This strategy has been used to improve the precision and interpretability of structure–function-behavior relationships in cognitive, perceptual, and clinical domains (Dougherty et al., 2005; Saygin et al., 2011, 2016; Reid et al., 2016; Lerma-Usabiaga et al., 2018; Yoshimine et al., 2018; Grotheer et al., 2019, 2022; Finzi et al., 2021; Kubota et al., 2023). However, it has historically been challenging to precisely link gray matter cortical regions with white matter bundles because they are comprised of separate tissue types that are qualified using different methods (fMRI and DWI respectively). Early studies that sought to link white matter bundles to function used approaches create spherically dilated regions of interest (ROIs) (Yeatman et al., 2013; Gomez et al., 2015) to extend functional regions into the white matter. However, these approaches can come at a cost to anatomical precision, especially when ROIs are small and close together. In recent years, advances in surface brain mapping and tractography have been used to maintain the spatial precision of the white matter associated with ROIs by projecting ROIs along the surface normal and restricting ROIs to the gray-matter-white-matter interface, putting ROIs in close proximity with the underlying white matter (Grotheer et al., 2019; Finzi et al., 2021; Kubota et al., 2023). These methods were developed and employed in the context of specific empirical studies, therefore, there is a need for an open-source software implementation for high throughput FSuB analysis. In addition, as detailed in this paper, several methodological factors relating to fMRI and DWI acquisition and processing are pertinent to the reliability, validity, and interpretation of the FSuB approach.

In the present article, we facilitate the adoption of the FSuB approach in four critical ways: (1) We provide suggestions and practical guidance for data acquisition and processing for extracting and analyzing FSuBs, with accompanying code. (2) We introduce the *FSuB-Extractor*, a flexible open-source software toolbox for producing and analyzing FSuBs. (3) We showcase a comprehensive FSuB workflow (beginning with raw data) and the *FSuB-Extractor* on a high-quality, publicly-available dataset, the Natural Scenes Dataset (Allen et al., 2022). (4) We validate the *FSuB-Extractor* against previously reported findings (Kubota et al., 2023). We hope this guide will allow for more accessible, user-friendly, and high-throughput FSuB analyses in future research, and hence contribute to a finer-grained understanding of the link between brain structure, brain function, and behavior.

## 2 MRI acquisition and processing suggestions for FSuB purposes

We note that the following guidelines may change as MRI acquisition and processing techniques evolve. These suggestions are derived from prior empirical work on MRI methods development and evaluation (e.g., Glasser et al., 2013; Daducci et al., 2014; Canales-Rodríguez et al., 2019; Esteban et al., 2019; Yeh et al., 2019; Grisot et al., 2021; Maffei et al., 2022). As detailed later in the text, the FSuB approach involves analyzing streamlines whose endpoints are proximal to functional ROIs in the gray matter. The following suggestions will help create well-made cortical surface reconstructions, white matter tractograms/bundles, and functional ROIs, which are needed to achieve optimal FSuB specificity.

### 2.1 Anatomical MRI acquisition

Anatomical images, such as T1- and T2-weighted images (T1w/T2w), should cover the whole brain and have a voxel size of no more than 1 mm isotropic, and preferably smaller if time allows (Glasser et al., 2014). At the minimum, a T1w anatomical image of the entire brain is usually required (although studies of some special populations, such as infants, may instead rely primarily on T2w images). Additionally collecting a T2w anatomical image enables one to better refine cortical surfaces in software packages such as *FreeSurfer* (Dale et al., 1999). Fat-suppressed T2w images can also be useful in correcting for echo-planar imaging (EPI) distortions (Wu et al., 2008; Irfanoglu et al., 2017; Montez et al., 2023). Thus, it is recommended to collect both T1w and T2w images, as long as it is practical to do so. Sequences with built-in motion correction, such as volumetric navigators (Tisdall et al., 2012) can reduce motion-related artifacts, leading to more reliable brain surface reconstructions, particularly in hyperkinetic populations such as children (Tisdall et al., 2016).

### 2.2 Anatomical MRI processing

**Figure 2:**
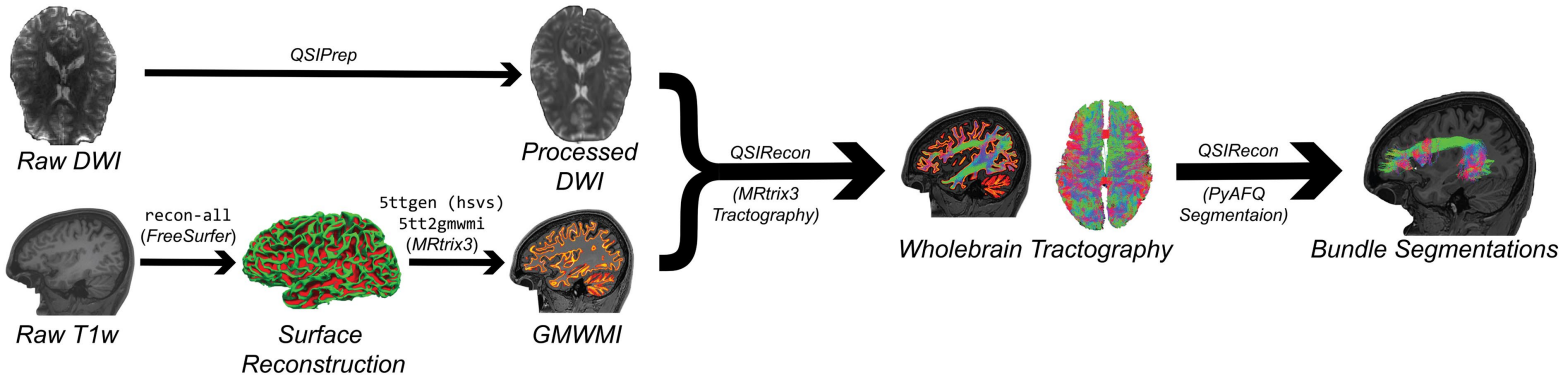
Simplified DWI (top) and anatomical (bottom) processing workflows implemented by the code in this article (Code Blocks 1 and 2). The result of these workflows is a set of bundle segmentations with streamlines reaching the gray matter white matter interface (GMWMI). Text in consolas font represents commands used in processing.

FSuB extraction works best when both functional regions of interest (fROI) and streamline endpoints can be defined nearest to the gray matter white matter interface (GMWMI). This allows one to minimize the search distance between fROI and streamline endpoints, mitigating concerns of false positive streamline inclusion. Information from brain surface reconstruction will lead to a more accurate GMWMI than relying on volumetric segmentation alone. *FreeSurfer* (Fischl, 2012), through the recon-all workflow, is the most common software solution for reconstructing the cortical surface. Although this is an automatic workflow, it is recommended to visualize outputs and manually correct defects (e.g., holes and handles) if it is practical to do so. A GMWMI can then be created using *MRtrix3*’s (Tournier et al., 2019) commands 5ttgen and 5tt2gmwmi (Figure 2). We recommend using the Hybrid Surface and Volume Segmentation (hsvs) algorithm, which leverages both surface and volumetric information from *FreeSurfer* (Smith et al., 2020).

### 2.3 DWI acquisition

DWI acquisitions should be chosen based on the planned fiber tracking algorithm and microstructural measures of interest. While both shelled schemes (e.g., high angular resolution diffusion imaging—HARDI) and cartesian grid schemes (e.g., diffusion spectrum imaging—DSI) may be used for tractography, some processing techniques, software, and microstructural measures may be uniquely suited for specific acquisition schemes. For example, a workflow based on constrained spherical deconvolution (Tournier et al., 2008; Jeurissen et al., 2014) will need data collected in spherical shells. Conversely, diffusion spectrum imaging (Wedeen et al., 2000) requires a cartesian grid sampling scheme. However, many models used to infer white matter microstructure, including those for diffusion tensor imaging (Basser et al., 1994), diffusion kurtosis imaging (DKI; Steven et al., 2014), generalized *q*-sampling imaging (GQI; Yeh et al., 2010), and neurite orientation dispersion and density indices (NODDI; Zhang et al., 2012), can be fit on either shelled or gridded schemes.

Regardless of one’s choice of sampling scheme, it is recommended to use DWI acquisitions that include both low (≤ 1,200 s/mm^2^) and high (≥ 2,000 s/mm^2^) *b*-values, which collectively yield fiber orientation distributions that better reflect more complex non-gaussian and hindered diffusion patterns (Assaf et al., 2004). This type of acquisition enables multi-compartmental signal modeling of fiber orientation distribution functions, which ultimately leads to better differentiation between tissue classes and more reliable white matter signal at tissue boundaries (Jeurissen et al., 2014). This type of acquisition also allows for using nuanced microstructural models, such as NODDI and DKI, and has benefits for tractography (Maffei et al., 2022). Stronger diffusion weighting emphasizes intra-axonal signal while attenuating extra-axonal signal (Huang et al., 2020). Thus, orientation distribution functions become more sharply tuned to better resolve crossing fibers. This leads to lower directional uncertainty in probabilistic tracking, and presumably better estimates of the primary diffusion direction in deterministic tracking (Setsompop et al., 2013). Additionally, acquiring a large number of DWI directions will yield a higher angular resolution scan for improved tractography, and we refer the reader to a set of recommendations for minimal number of diffusion directions (Schilling et al., 2022). A typical minimal DTI acquisition, with a single low *b*-value shell at few (≤ 32) directions, is not recommended, since it has low angular resolution, is unable to take advantage of multiple *b*-value DWI signal modeling, does not have high *b*-valued shells, and restricts microstructural measures primarily to DTI metrics such as fractional anisotropy and mean diffusivity.

Additionally, susceptibility distortion correction (SDC) should be applied to DWI images to correct for geometric distortion artifacts present in fast EPI acquisitions (Andersson et al., 2003). SDC leads to better anatomical correspondence not only between T1w and DWI images, but also between DWI and fMRI images, assuming both images have undergone SDC. There are multiple ways to enable SDC in DWI. One method is to collect a pair of DWI acquisitions that have opposing phase encoding directions (Andersson et al., 2003; Irfanoglu et al., 2015). This option has the largest time cost, but has the advantage of gathering multiple data points per direction, which can mitigate the impact of noise. Another is to collect a dedicated field map before DWI scans to quantify magnetic susceptibility distortions. Additionally, if one is using a dataset that has missing or low-quality field maps, one can use field map-less distortion correction methods such as *Synb0-DisCo* (Schilling et al., 2019a).

### 2.4 DWI processing

#### 2.4.1 DWI preprocessing

We recommend using automated, flexible, and robust preprocessing softwares, such as *QSIPrep*^1^ (Cieslak et al., 2021), for ease of operation and reproducibility. Regardless of what software is used, the following preprocessing steps are suggested as a minimum:

1. Denoising. For example, the *MRtrix3* software package (Tournier et al., 2019) implements Marchenko-Pastur principal component analysis (Veraart et al., 2016b) via dwidenoise, and the *DIPY* software package (Garyfallidis et al., 2014) can perform the self-supervised *patch2self* method (Fadnavis et al., 2020).
2. Susceptibility distortion correction, such as *FSL*’s (Jenkinson et al., 2012) topup functionality (Andersson et al., 2003). If using *Synb0-DisCo*, please refer to their GitHub repository for usage instructions.^1^
3. Eddy current and motion correction (often done simultaneously). For example, *FSL* implements this with the eddy command (Andersson and Sotiropoulos, 2016), and the *TORTOISE* toolbox (Irfanoglu et al., 2017) includes this in their DIFFPREP module.
4. Gibbs deringing (Veraart et al., 2016a), which will mitigate DWI artifacts at the interfaces between white matter and other neural compartments. This can be implemented in *MRtrix3* with mrdegibbs, which is based on a local sub-voxel shift approach (Kellner et al., 2016) and is well-suited for full Fourier acquisitions. *TORTOISE* includes a Gibbs deringing method that is better suited for partial Fourier acquisitions (Lee et al., 2021).

Additionally, the DWI image and T1w image need to be aligned to one another. While reorienting DWI images should be minimized and done in a single step due to concerns of data interpolation and the need to correct the gradient table (Leemans and Jones, 2009), we note that it is common in image preprocessing pipelines to rotate derivatives such that the anterior and posterior commissures are at the same level (commonly referred to as ACPC alignment). This reorientation enforces common image orientations and origins across subjects, which benefits downstream quality control and image registration if needed. In ACPC-aligned DWI images for example, a directionally-colored FA map should always indicate left-to-right orientation (typically bright red) in the corpus callosum, and visualizing these maps is a quick quality assurance check that the gradient table is valid. The same map in an image with rotational bias not ACPC aligned might not have as distinctive of a color in the corpus callosum, which could confound quality control. As long as SDC is applied to the DWI image, only linear warping should be necessary to make this alignment (Chen et al., 2019).

Upsampling the DWI image to 1.25 mm isotropic voxels, if needed, e.g., with *MRtrix3’*s mrresize, can be useful creating a more resolvable border between gray and white matter (Dyrby et al., 2014). However, this may only provide meaningful benefits if the images were acquired with only slightly larger voxels (e.g., 1.5 mm), and may otherwise increase computational burden with trivial benefits. For FSuB purposes, in which regions of interest may be defined on anatomical surfaces, upsampling beyond the resolution of the T1w image will provide diminishing returns.

#### 2.4.2 DWI postprocessing

After preprocessing, steps should be taken towards producing a whole-brain tractogram and/or bundle segmentations. Some bundle segmentation algorithms will operate on a whole-brain tractogram based on streamline clustering, such as *Recobundles* (Garyfallidis et al., 2018), or anatomical waypoint criteria, such as *PyAFQ* (Yeatman et al., 2012; Kruper et al., 2021). Other segmentation algorithms, including *TractSeg* (Wasserthal et al., 2018) and *DSI-Studio’s* AutoTrack (Yeh et al., 2018), can directly segment bundles using the DWI signal or modeled signal without starting from a whole-brain tractogram. The precise steps one may take to identify bundles may vary based on one’s software preference and acquisition scheme. For example, *TractSeg* and many of *MRtrix3*’s tractography implementations require derivatives from constrained spherical deconvolution, which necessitates a shelled acquisition.

Regardless of which method is chosen, it is important that the resulting streamlines reach the GMWMI to minimize the distance between streamline endpoints and gray matter ROIs. Minimizing the search distance between streamline endpoints and fROIs mitigates concerns of false positive streamline inclusion when defining FSuBs. To this end, *MRtrix3* can implement anatomically constrained whole-brain tractography (Smith et al., 2012), which enables precise streamline cropping at the GMWMI, seeding on the GMWMI, as well as backtracking which removes streamlines with anatomically implausible ends (i.e., within non-superficial white matter). A similar technique that can be run in *DIPY* is imposing a Continuous Map Criterion (CMC) based on tissue probability maps (Girard et al., 2014). Surface tractography, or seeding directly on the surface meshes, can also be achieved through *DIPY* through surface-enhanced tragography (St-Onge et al., 2018) and *FSL* (Jenkinson et al., 2012; Warrington et al., 2020). Visualizing the streamlines over the T1w image is recommended for quality assurance.

Since cortical regions of interest may be small, we recommend that tractograms and bundles be sufficiently dense, e.g., 5-10 million streamlines for whole-brain coverage. Although such dense whole-brain tractograms are prone to false positives (Maier-Hein et al., 2017), strategies exist to mitigate this. These mechanisms use the underlying DWI signal or fiber orientation distributions to make judgements about streamline validity. This can result in streamlines being removed if they are redundant or biologically implausible (Smith et al., 2013), or alternatively given weights corresponding to their estimated contributions to the DWI signal (Pestilli et al., 2014; Daducci et al., 2015; Smith et al., 2015). Both of these strategies have their own advantages and use cases. Removing streamlines can increase computational efficiency and be a valid approach if one’s downstream analyses cannot incorporate streamline weights. Using streamline weights can allow one to analyze a sufficiently dense tractogram while biasing against implausible streamlines, and summation of these weights in bundles can be an informative measure of structural connectivity (Smith et al., 2022). Note that these strategies are only valid when applied to a whole-brain tractogram.

An alternative strategy is to create FSuBs by only seeding and terminating streamlines between a pair of ROIs. Advantages of this approach are that it can be less computationally intensive compared to generating a whole-brain tractogram, and it allows one to explicitly control the number of final streamlines in the FSuB (which we note is *not* the same as tract volume). However, we do not recommend this approach, as this method does not ensure that the resulting FSuB are derived from a canonical bundle (additional waypoints or exclusion masks could help in this regard, however). Additionally, streamline algorithms that detect false positives require a whole-brain tractogram to reliably estimate streamline contributions to the underlying DWI signal (Smith et al., 2022). So, FSuBs created by directly seeding between fROIs cannot take advantage of these mechanisms.

### 2.5 fMRI acquisition

Although the *FSuB-Extractor* can accept gray matter ROIs defined by any criteria, we anticipate the most common use case will be to input fROIs that are defined from task-based fMRI analyses (e.g., areas of high activation from a statistical parametric map). Due to the variable nature of fMRI tasks, it is difficult to prescribe best guidelines for fMRI acquisitions, as these could vary based on the task and the effect one is trying to resolve. However, in general for FSuB purposes it is best to strive for the smallest voxel resolution one can achieve while maintaining an acceptable signal-to-noise ratio (SNR), which will increase the anatomical precision of functional clusters. Since higher voxel resolutions and shorter TRs come at the expense of lower SNR, number of TRs and acquisition parameters should ultimately be decided based on the SNR, the size of fROIs, and the expected task effect size (Murphy et al., 2007). Similar to DWI acquisitions, field maps should be collected before fMRI scans to perform SDC, and field map-less distortion methods are available as well (for example, *SynBOLD-DisCo*; Yu et al., 2023).

To ensure that the FSuBs are specific to the function being studied, a well-designed localizer task should be used. Localizers typically aim to identify regions of the brain that are active during a target condition/task compared to control conditions/tasks. For example, a language localizer focused on semantic processing may contrast perceiving intact vs. degraded speech (Scott et al., 2017), and a localizer identifying high-level visual areas might contrast responses to one category of stimuli (e.g., faces) compared to many others (e.g., words, places, bodies, objects) (Stigliani et al., 2015). Note that in both cases, low-level features of the stimuli (speech sounds and visually-presented images) are held constant, so that it is possible to identify neural responses that are specific to the manipulation of interest (in this case, language comprehension or face perception). Regardless of the task being studied, it is important to consider both the target and control conditions. A localizer that contrasts responses to faces compared to checkerboards, for example, may result in a broader and less functionally precise region than a localizer that contrasts responses to faces with a variety of other visual categories. It is also important to consider the limitations of interpreting localizer results. Just because a region responds more to faces compared to other stimuli included in the localizer does not mean that the region is not responsive to other categories or conditions that were not included in the experiment.

### 2.6 fMRI processing

#### 2.6.1 fMRI preprocessing

We similarly recommend using automated, flexible and robust preprocessing software, such as *fMRIPrep*^3^ (Esteban et al., 2019) for ease of operation and reproducibility. Regardless of what software is used, the following preprocessing steps are suggested as a minimum:

1. Motion correction
2. Susceptibility Distortion Correction (SDC)
3. Alignment to T1w image

There are several reasons to prefer conducting fROI analyses on the cortical surface of the brain, as opposed to volumetric analysis. Surface-based analysis has the advantage of producing statistical maps that are specifically conformed to the brain’s geometry. In volumetric analysis, there is often a temptation to dilate fROIs to project them into white matter, which sacrifices anatomical specificity. For example, dilating a fROI may extend the fROI to include a sulcus that is nearby in volumetric, but not surface space. Therefore, for FSuB analysis, we strongly recommend using surface-based methods. Surface-based methods are not as ubiquitous or as standardized as volumetric analyses though, further warranting increased documentation. For these reasons, the code examples below are particularly tailored towards working with surface data. However, we include volumetric analysis code in Supplementary material S4, and the *FSuB*-*Extractor* is compatible with both volumetric and surface-based fROIs. Many of the methodological considerations discussed below also apply to volumetric analysis.

#### 2.6.2 The decision to smooth

After preprocessing, steps should be taken towards running general linear models (GLM) to produce statistical maps that highlight where the brain is specialized for the task (Friston et al., 1994). A user must decide whether to smooth their data. Smoothing data increases SNR at the expense of anatomical specificity. We advise against smoothing data in volumetric space for FSuB analysis, as neighboring voxels can include relatively distant regions on the cortical surface due to complexities of cortical folding patterns (Weiner and Grill-Spector, 2013; Brodoehl et al., 2020). Whether one should smooth data for surface-based analysis is dependent on a few factors of the analysis. If one is interested in small fROIs, smoothing may overly blur statistical maps, confounding localization of fROIs. Another important consideration in the decision to smooth is if fROIs will be identified automatically or manually, as described in more detail in the following sections. Smoothing data may be more appropriate when using automated statistical thresholding to define fROIs. The smoothed statistical maps will tend to have higher more-distributed effect sizes so the resulting masks could be more continuous, leading to a more contiguous FSuB bundle core. However, smoothing is not necessary, and is more likely detrimental, when manually drawing fROIs due to blurring of precise functional boundaries. Most of the extant FSuB literature does not smooth fMRI images (Grotheer et al., 2019; Kubota et al., 2023).

#### 2.6.3 Running a surface-based GLM

GLMs can be used to produce statistical maps for one’s functional contrast of interest, as well as denoise data (by including nuisance regressors). There is no standard recommendation on what regressors to choose for denoising the data (Mayer et al., 2019). An example regression basis may include head-motion parameters (rotation and translation in the X, Y, and Z directions), some physiological noise regressor [e.g., mean signal outside of gray matter or ACompCor components (Behzadi et al., 2007)], and frame censoring for non-steady state volumes. Regressors should be chosen based on factors such as the quality of the data, as well as the temporal degrees of freedom one is comfortable with sacrificing (Mayer et al., 2019).

#### 2.6.4 Identifying functional regions of interest (fROI)

**Figure 3:**
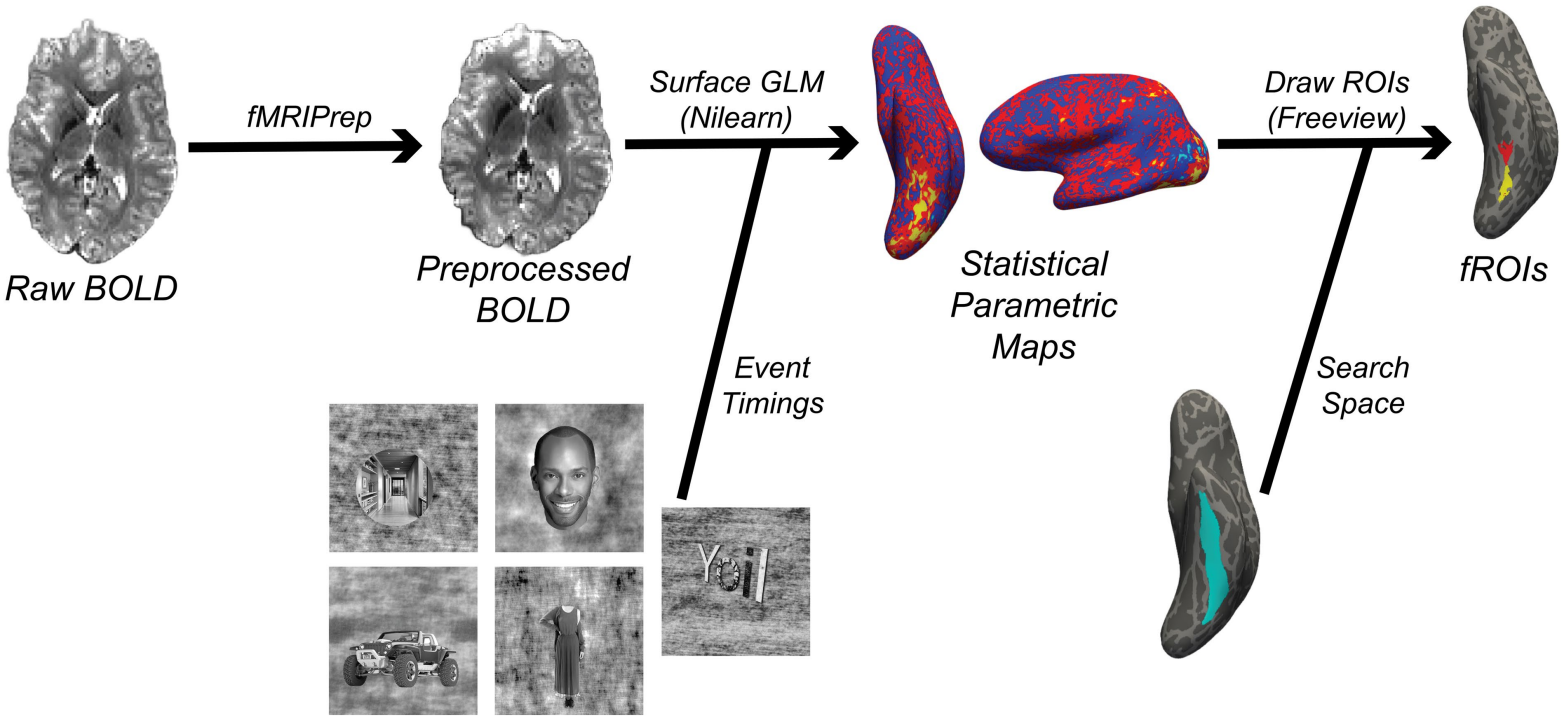
Simplified functional MRI processing workflow implemented by the code in this article (Code Block 3). The result of this workflow is a set of functional ROIs (fROIs) that are used in the FSuB extraction process. The statistical parametric map and fROIs in this figure are derived from a contrast comparing responses to character vs. all other stimuli categories. Two fROIs are drawn for character-selective regions in the mid-occipitotemporal sulcus (red) and posterior occipitotemporal sulcus (yellow), within the larger occipitotemporal sulcus (light blue).

Manually drawing masks involves having a user outline fROIs on the brain based on visualizing statistical maps, while automated statistical methods will create fROIs based on thresholding statistical maps. In either case, fROIs should come from a pre-defined search space that is consistent across subjects (Figure 3). This may be defined anatomically, for example by a given landmark such as a specific sulcus (Weiner et al., 2014). A functionally-specific alternative (albeit not mutually exclusive) is to define search spaces based on group-level contrast maps (Nieto-Castañón and Fedorenko, 2012). This approach requires two runs of a localizer task (one that is used on the group-level to define the search space, and one to find each subject’s fROI within that search space). This approach also necessitates data first being warped to a standard space, after which the resulting search space should be brought back to subject space. In surface-based analyses, this space could be *FreeSurfer*’s fsaverage space, or the fsLR space that is used by projects such as the Human Connectome Project (Glasser et al., 2013), while MNI space is the most common standard space for volumetric data. We note that one should only move to a common space if necessary, and all pertinent transformations should be combined and applied in a single step to minimize data interpolation (Wang et al., 2022). Otherwise, fROI determination should remain in subject-space to minimize data interpolation and ensure better anatomical correspondence to DWI derivatives.

It is important that criteria for defining neural selectivity is consistent across subjects. This may be defined as everywhere in a search-space where a contrast test statistic is greater than some value [e.g., *t*-value >3 (Nordt et al., 2021), or *p*-value less than 0.001 (Kanwisher et al., 1997)], or the top X% area with the highest effect sizes [e.g., top 10% of *t-*values (Scott et al., 2017)]. One should be cognizant of the biases these thresholding methods can introduce (Nieto-Castañón and Fedorenko, 2012). For example, if one defines an fROI as all voxels or vertices in a given space that exceed a particular *t*-statistic, subjects will likely have different sized masks, regardless of whether you are automating fROI definition, or drawing ROIs. Thus, an FSuB summary metric such as streamline count or volume would be affected by size biases, as a larger ROI inherently has more connected streamlines. One could control for this *post-hoc* by normalizing metrics by fROI volume, although consideration should be given as to how this changes the interpretation of the results. While the alternative automated strategy of selecting the top X% area controls for fROI size, it introduces the possibility of including areas that are not particularly active for the contrast if the threshold is defined too liberally. Careful exploration should be used to determine the best fROI determination strategies for addressing the given hypothesis. In general, if it is practical to do so and does not create critical biases, we recommend identifying fROIs by manually drawing over unsmoothed statistical maps for the highest degree of functional and anatomical precision. We present a guide to drawing fROIs in *Freeview* (the image viewer included with *FreeSurfer*) in Supplementary material S5.

## 3 FSuB workflow tutorial and software

For succinctness and generalizability, our processing code examples are based primarily on the Brain Imaging Data Structure (BIDS) standard and BIDS applications (Gorgolewski et al., 2016, 2017), though other software and pipelines may be suitable as well. After minimal changes to the code examples below, processing should work on most BIDS-valid neuroimaging datasets. The *FSuB-Extractor* is not limited to working with preprocessed derivatives from BIDS applications, and as the BIDS standard changes and software are updated, the following code may need to be modified accordingly. Arguments in the code may have to be added or altered for specific use cases, so we encourage readers to explore the documentation of the software we discuss here. Our examples use Unix-like syntax, which is standard on Linux and Macintosh machines as some dependencies of the software used are not officially supported by Windows. Paths to files or directories will have to be changed to match the user’s filesystem. Many workflows described below are executed in software containers. We present the syntax for Singularity/Apptainer (Kurtzer et al., 2017), since it is standard for research-grade high-performance computer clusters, but Docker (Merkel, 2014) can also be used after making minimal adjustments to the syntax (see Supplementary material S1). Singularity/Apptainer containers can be built using this example command: singularity build fmriprep_23.2.0a2.img docker://nipreps/fmriprep:23.2.0a2, where repository, software, and version names are derived from the software’s DockerHub web page.

Below, we use data from the Natural Scenes Dataset (NSD) (Allen et al., 2022), which is openly accessible through http://naturalscenesdataset.org/ and has a BIDS-valid distribution. This dataset includes high quality anatomical scans, high angular resolution DWI, field maps, and high-resolution fMRI data in eight participants. The functional visual category localizer task (“floc”) presents participants with different visual stimulus categories including characters, bodies, faces, places, and objects (Stigliani et al., 2015), and is widely used to identify functional regions selective to different categories in individual subjects’ brains (Stigliani et al., 2015; Margalit et al., 2020; Finzi et al., 2021). More detailed acquisition parameters may be found in the dataset descriptor publication (Allen et al., 2022). We share a minimal BIDS-valid dataset which contains one subject’s data. This, along with code and derivatives for the processes below, may be found at https://osf.io/zf5q7/. We note that the NSD dataset includes preprocessed derivatives, which may be otherwise preferred if analyzing NSD data for consistency with other studies. From the preprocessed data, we only use their precomputed cortical surface reconstructions, since they have been manually corrected by experts. All figures within this manuscript, unless otherwise noted, depict derivatives from the first subject of the NSD dataset (“sub-01”) as processed by the code presented in this guide.

### 3.1 MRI processing example

#### 3.1.1 Anatomical preprocessing

The code in Code Block 1 invoke *sMRIPrep* (Esteban et al., 2023)^4^, a structural MRI preprocessing BIDS application which includes recon-all in the pipeline. The proceeding code will also create the GMWMI from the *FreeSurfer* outputs. However, we note that these processes can also be performed as part of the fMRI and DWI workflows described later (as well as part of *FSuB-Extractor*), so it is not necessary to run this separately.

**Code Block 1: Code for preprocessing structural MRI data with *FreeSurfer* and *MRtrix3*.**

*#!/bin/bash -l*
*## Define important paths and names*
bids="/path/to/nsd_bids/" *# Or replace with your own BIDS dataset*
workdir="/path/to/scratch/space/" *# e.g., /tmp*
smriprep_IMG="/path/to/smriprep_container.img" *# Software container*
fs_license="/path/to/freesurfer/license.txt" *# FreeSurfer license*
subject="sub-01" *# Or replace with your own subject ID*

*## Run sMRIPrep*
singularity run --containall -e \
 *# Can also use Docker*
  -B ${bids},${workdir},${fs_license} \
  ${smriprep_IMG} ${bids} ${bids}/derivatives participant \
  -w ${workdir} \ *# Scratch directory*
 --participant-label ${subject} \ *# Remove argument to process everyone in data set*
  --fs-license-file ${fs_license} *# FreeSurfer license file*

*## Make the GMWMI using MRtrix3*
export SUBJECTS_DIR="${bids}/derivatives/freesurfer/" *# Where to find FS outputs*
gmwmi_outdir="${bids}/derivatives/gmwmi_example/${subject}/"
mkdir -p ${gmwmi_outdir}
5ttgen hsvs ${SUBJECTS_DIR}/${subject}/ ${gmwmi_outdir}/${subject}_desc-5tt.nii.gz \
   -scratch ${workdir} *# Generate a 5-tissue-type segmentation image*
5tt2gmwmi ${gmwmi_outdir}/${subject}_desc-5tt.nii.gz \
   ${gmwmi_outdir}/${subject}_desc-5tt.nii.gz *# Make GMWMI*

#### 3.1.2 DWI processing

After installation, the *QSIPrep* (Cieslak et al., 2021) command Code Block 2 (based off of version 0.19.1) will perform all of the recommended DWI preprocessing steps, presuming one is working with a BIDS-valid dataset with files needed to run SDC as described earlier. It then creates an anatomically-constrained tractogram with 10 million streamlines which is subsequently segmented into bundles with *PyAFQ* (Yeatman et al., 2012; Kruper et al., 2021) (Figure 2). Metrics from NODDI and diffusion kurtosis imaging are also calculated. The post-processing reconstruction specification can be found in the associated OSF repository. Additional pre-defined post-processing pipelines are available in *QSIPrep* as well.^5^ We refer the reader to item S6 in Supplementary materials for a full description of processing steps performed by *QSIPrep*.

**Code Block 2: Code for pre- and postprocessing DWI data with *QSIPrep*.**

*#!/bin/bash -l*
*## Define important paths*
bids="/path/to/nsd_bids/" *# Or replace with your own BIDS dataset*
workdir="/path/to/scratch/space/" *# e.g., /tmp*
qsiprep_IMG="/path/to/qsiprep_container.img" *# Software container*
subject="sub-01" *# Or replace with your own subject ID*
recon_spec="${bids}/code/qsiprep/recon_spec.json" *# Post-processing pipeline json specification*

*## Run QSIPrep*
singularity run --containall -e \ *# Can also use Docker*
   -B ${bids},${workdir} \
    ${qsiprep_IMG} ${bids} ${bids}/derivatives participant \
   --participant-label ${subject} \ *# Remove argument to process everyone in data set*
    -w ${workdir} --output_resolution 1.25 \ *# Upsample data to 1.25mm*
    --unringing-method mrdegibbs \ *# Can also choose rpg from TORTOISE*
   --pepolar-method DRBUDDI \ *# Can also choose TOPUP from FSL*
   --denoise_method patch2self \ *# Can also choose dwidenoise from MRtrix3*
   --freesurfer-input ${bids}/derivatives/freesurfer \ *# FreeSurfer outputs*
   --fs-license-file ${fs_license} \ *# FreeSurfer license file*
   --recon-spec ${recon_spec} *# Reconstruction pipeline name/JSON file*

While this software is comprehensive and convenient, we note that *QSIPrep* will rotate its derivatives such that the anterior and posterior commissures are at the same level (commonly referred to as ACPC aligned). Therefore, an fROI derived from a different anatomical space (e.g., *FreeSurfer* surface or native T1w space) will need to be aligned to the DWI image. The transformation from volumetric native-to-ACPC space is saved by *QSIPrep* by default. Additionally, in Supplementary materials we share code to calculate the registration between *FreeSurfer* and *QSIPrep*-derived anatomical images (Supplementary material S2). The *FSuB-Extractor* also has the functionality to perform this registration, when provided with a T1w image and brain mask aligned to the DWI derivatives.

#### 3.1.3 fMRI processing

After installation, the example *fMRIPrep* (Esteban et al., 2019) command in Code Block 3 based off of version 23.2.0a2, will perform recommended fMRI preprocessing steps, presuming one is working with a BIDS-valid dataset with files needed to run SDC as described earlier. We refer the reader to item S6 in Supplementary materials for a full description of processing steps performed by *fMRIPrep*.

**Code Block 3: Code for fMRI preprocessing with fMRIPrep.**

*#!/bin/bash -l*
*## Define important paths*
bids="/path/to/nsd_bids/" *# Or replace with your own BIDS dataset*
workdir="/path/to/scratch/space/" *# e.g., /tmp*
fs_license="/path/to/freesurfer/license.txt" *# FreeSurfer license*
fmriprep_IMG="/path/to/fmriprep_container.img" *# Software container*
subject="sub-01" *# Or replace with your own subject ID*

singularity run --containall -e \ *# Can also use Docker*
   -B ${bids},${workdir},${fs_license} \
   ${fmriprep_IMG} ${bids} ${bids}/derivatives participant \
   --participant-label ${subject} \ *# Remove argument to process everyone in data set*
   -w ${workdir} \ *# Scratch / working directory*
   --fs-license-file ${fs_license} \ *# FreeSurfer license file*
   --fs-subjects-dir ${bids}/derivatives/freesurfer \ *# Where to look for and store FreeSurfer recon-all outputs*
   --output-spaces T1w fsnative MNI152NLin2009cAsym \ *# Native space volumetric and surface outputs, but MNI can be useful for quality assurance and visualization*
   --slice-time-ref 0 \ *# Some software assume slice time is corrected to TR start*
   --cifti-output 91k \ *# If you want common-space surface outputs*
   --project-goodvoxels \ *# Don’t project high-variance voxels to surface*

If one chooses to smooth the data, the code in Code Block 4 will smooth the BOLD surface outputs at a specified gaussian kernel size.

**Code Block 4: Code for smoothing surface fMRI data.**

*#!/bin/bash -l*
bids="/path/to/nsd_bids/" *# Or replace with your own BIDS dataset*
subject="sub-01" *# Or replace with your own subject ID*
task="floc" *# Which task to process*
declare -a hemis=("L" "R") *# Which hemispheres to process*
declare -a runs=("1" "2" "3" "4" "5" "6") *# Which runs to process*
space="fsnative" *# Using native space surface outputs*
fwhm="4" *# Desired smoothing kernel size (mm FWHM)*
*# Where to find data*
fmriprep_dir=${bids}/derivatives/fmriprep/
freesurfer_dir=${bids}/derivatives/freesurfer/
export SUBJECTS_DIR=${freesurfer_dir} *# Tell FreeSurfer where subjects live*

*# Loop over hemispheres*
**for** hemi **in** ${hemis[@]}; **do**
  **if** ["$hemi" == "L" ];
       **then** hemi_fs="lh"; *# Hemi name in FreeSurfer conventions; "lh" or "rh"*
  **elif** [ "$hemi" == "R" ];
       **then** hemi_fs="rh"; *# Hemi name in FreeSurfer conventions; "lh" or "rh"*
  **fi**;

   *# Loop over runs*
   **for** run **in** ${runs[@]}; **do**
      *# Define input and output names*
gii_in="${fmriprep_dir}/${subject}/func/${subject}_task-${task}_run-${run}_hemi-${hemi}_space-${space}_bold.func.gii" *# Input file name*
      gii_out=${gii_in/_bold/_desc-smoothed_bold} *# Add smoothed label for output name*

    *# Perform the smoothing*
    mris_fwhm --i ${gii_in} --o ${gii_out} --so \
       --fwhm ${fwhm} --subject ${subject} --hemi ${hemi_fs}
  **done**
**done**

We present a code example that uses *Nilearn* (Abraham et al., 2014) to run GLMs on multiple runs of surface data, producing run-specific and session-averaged statistical maps. Due to its length, this function is not presented in the main text but can be found in Supplementary material S3 and the associated OSF repository.

As an example of an automated thresholding workflow, which numerically thresholds a statistical map to determine fROIs, the code in Code Block 5 extracts the 10% of vertices with the highest character-selective contrast *z*-score in the left mid-occipitotemporal sulcus (mOTS). This can be adapted for other search spaces and threshold values. This code requires *FreeSurfer* and *Connectome Workbench*^6^ to be installed.

**Code Block 5: Workflow for statistically thresholding functional ROIs.**

*#!/bin/bash -l*
*## Define important paths and parameters*
bids="/path/to/nsd_bids/" *# Replace with your BIDS directory*
freesurfer_dir=${bids}/derivatives/freesurfer/ *# FreeSurfer outputs*
export SUBJECTS_DIR=${freesurfer_dir} *# Tell FreeSurfer where subjects live*
l1_gifti=${bids}/derivatives/l1_gifti/ *# Where statmaps from surface GLM code are*
subject="sub-01" *# Subject name*
hemi="L" *# Hemisphere name in fMRIPrep naming convention*
space="fsnative" *# Space of statmaps*
contrast="chratactersGTother" *# GLM contrast name*
stat="z" *# Which stat to threshold*
smoothed_label="desc-smoothed_" *# (leave blank if not smoothing)*
outdir=${bids}/derivatives/threshold_fROIs/${subject} *# Where outputs will go*
mkdir -p ${outdir} *# Make the output directory*
region_label="/path/to/lh.ots.label" *# A FS label file that defines the searchspace*
label_name="ots" *# A descriptive name for the label*
value_percentile="90" *# Percentile to threshold statistic*

*# Locate the statistical map*
statmap=${l1_gifti}/${subject}/${subject}_hemi-${hemi}_space-${space}_contrast-${contrast}_stat-${stat}_${smoothed_label}
statmap.func.gii *# Path to statmap*

*## Get hemisphere name in FreeSurfer naming convention*
**if** [ "$hemi" == "L" ];
  **then** hemi_fs="lh"; *# Hemi name in FreeSurfer conventions; "lh" or "rh"*
  **elif** [ "$hemi" == "R" ];
  **then** hemi_fs="rh"; *# Hemi name in FreeSurfer conventions; "lh" or "rh"*
**fi**;

*## Convert FS label to GIFTI*
region_gii=${outdir}/${subject}_hemi-${hemi}_space-${space}_desc-${label_name}_roi.func.gii
mris_convert --label ${region_label} ${label_name} \
    ${freesurfer_dir}/${subject}/surf/${hemi_fs}.white ${region_gii}

*## Mask statmap by label*
masked_statmap=${outdir}/${subject}_hemi-${hemi}_space-${space}_contrast-${contrast}_stat-${stat}_desc-${label_name}_desc-masked_roi.func.gii
 *# Output variable for next command*
wb_command -metric-mask ${statmap} ${region_gii} ${masked_statmap}

*## Find threshold corresponding to top X% of values in ROI, save value as "thresh"*
wb_command -metric-stats ${statmap} -roi ${region_gii} -percentile ${value_percentile} | read thresh
echo "${value_percentile} percentile of ${stat} statistic within ${hemi} ${label_name} is ${thresh}"

*## Make a binary mask of values above that threshold*
*# Output variable for next command*
thresholded_statmap=${outdir}/${subject}_hemi-${hemi}_space-${space}_contrast-${contrast}_stat-${stat}_desc-${label_name}_desc-masked_desc-thresholded_roi.func.gii
*# Binarize and threshold the statmap*
wb_command -metric-math "(statmap > ${thresh})" ${thresholded_statmap} \
    -var statmap ${masked_statmap}

### 3.2 The *FSuB-Extractor*

The *FSuB-Extractor*^7^ is a flexible open-source toolbox for extracting and analyzing FSuBs. By the time one is ready to run the software, one should have *at least* the following data, which can be produced following the instructions above:

1. *FreeSurfer* recon-all outputs derived from the subject’s anatomical image(s).
2. A tractogram object (either a specific bundle or whole-brain) in .tck or .trk format.
3. One or two fROIs in .nii.gz or *FreeSurfer* .gii/.label/.annot formats.

The software provides a general framework to identify the white matter connections of a given gray matter region, while also providing flexibility for different analysis paths. For example, users can decide how to define their fROIs and also which software to use when segmenting bundles [e.g., *AFQ, TractSeg, XTRACT, DSI-Studio* (Wasserthal et al., 2018; Warrington et al., 2020; Kruper et al., 2021)]. By default, all inputs are presumed to be aligned to one another, but one can optionally supply a registration between the *FreeSurfer* outputs and DWI outputs if they have different alignments (*QSIPrep* aligns outputs to the ACPC line, for example).

Full installation, usage instructions, and documentation of inputs and outputs can be found on the wiki in the GitHub repository.^8^ Any bugs or questions can be addressed by posting an issue in the software repository.^9^ Below, we outline the primary workflow of the *FSuB-Extractor* and provide example code (Code Block 6) to work with outputs of the processes detailed above. Additionally, in Supplementary materials, we validate our software by showing how it achieves comparable results to custom in-house FSuB code used in Kubota et al. (2023) (Supplementary material S5).

**Figure 4:**
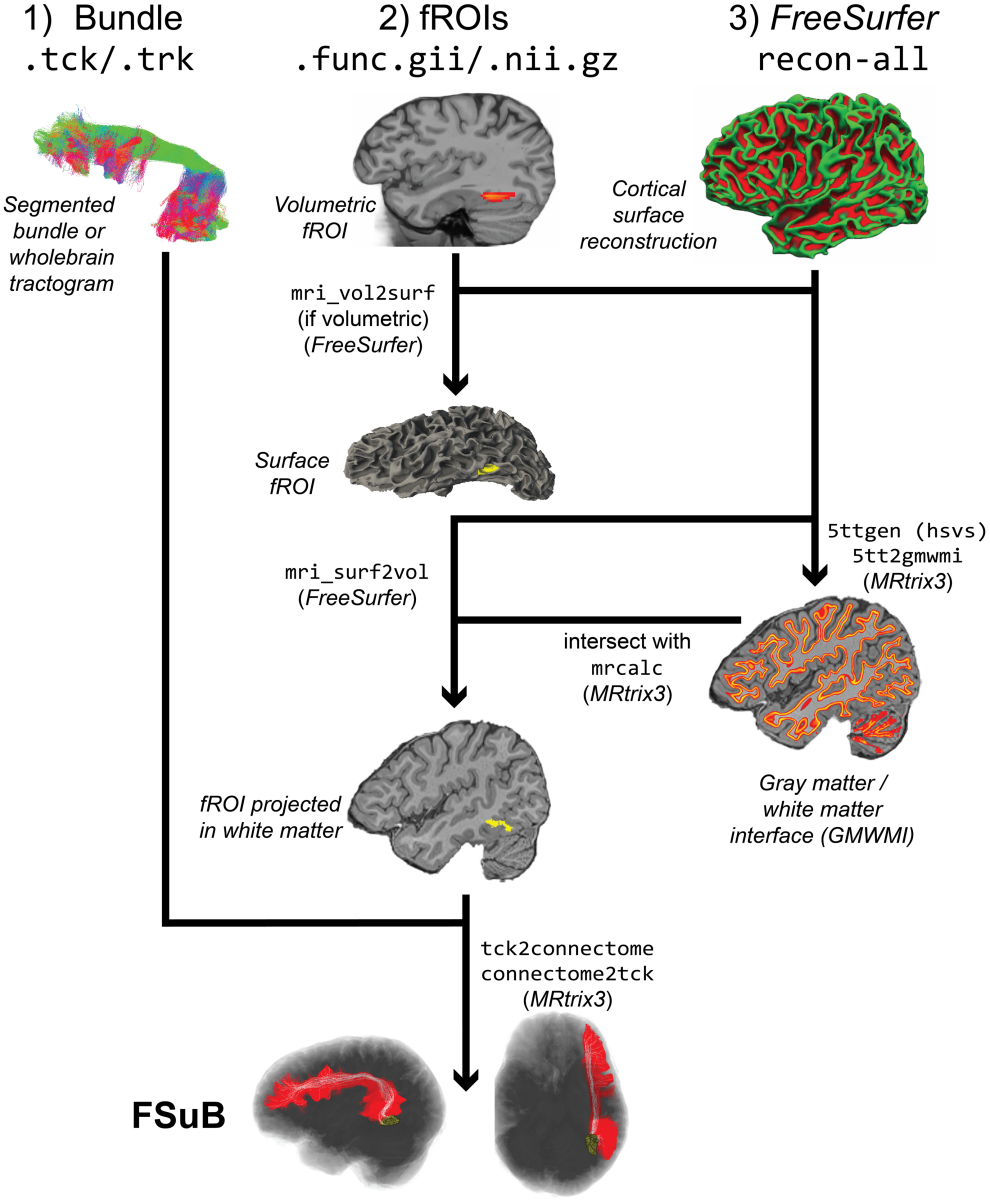
FSuB-Extractor workflow (Code Block 6). The left, middle, and right columns track how DWI, fMRI, and anatomical data are used in the workflow, respectively. The depicted workflow showcases the most basic functionality of the software. The final FSuB is denoted by the white streamlines, within the larger original bundle in red.

The *FSuB-Extractor* automates the following steps (Figure 4):

1. Gray Matter White Matter Interface (GMWMI) Creation: The GMWMI represents the border between white and gray matter. To make a GMWMI, a 5 tissue-type (5tt) image is first created with *MRtrix3*’s 5ttgen using the Hybrid Surface Volume Segmentation algorithm (Smith et al., 2020), which incorporates *FreeSurfer* surface reconstructions. From this image, a probabilistic GMWMI is extracted with 5tt2gmwmi, which is binarized at an adjustable threshold. A separately made 5tt image may also be passed into the function to expedite this process.
2. fROI Projection: In order to intersect fROIs in the gray matter and white matter endpoints, fROIs are projected onto the GMWMI. While one could simply dilate fROIs until they reach the GMWMI, such radial expansion will overestimate the size of the fROI. Projection to the cortical surface is done with *FreeSurfer*’s mri_vol2surf (this is only applied for volumetric fROIs and is skipped for surface-based fROIs). The resulting mask is projected into the GMWMI and converted back to a NIFTI file for streamline matching with mri_surf2vol. Parameters of projection can be set in the command line.
3. fROI-GMWMI Intersection. The streamline search space is defined as the intersection of the projected fROIs and GMWMI. This process is performed by *MRtrix3*’s mrcalc by multiplying the two masks together.
4. Converting .trk Streamlines to .tck (as needed): For compatibility with *MRtrix3* tools, input streamlines are converted to .tck.
5. Streamline-fROI matching: A combination of *MRtrix3* commands, tck2connectome and connectome2tck, are used for streamline filtering and creating the FSuB. All of the streamline matching criteria options that *MRtrix3* provides (e.g., a radial search from the streamline endpoint or a forward search) can be used, with parameters that can be defined in the command line. Considerations for these options are discussed in (Yeh et al., 2019), and our default value of a 2 mm radial search is adopted from that article. The streamlines matched to the fROI(s) are saved out as the FSuB. If two fROIs are input to the *FSuB-Extractor*, streamlines that connect the two fROIs are saved out as the FSuB.
6. Visualization: A visualization of the fROI(s), original bundle, and FSuB, can be saved out, with image parameters (e.g., fROI and bundle colors) adjustable at the command line. This visualization can also be interactive, enabling the user to examine the results from all angles.

All primary outputs and intermediate files are saved out in a BIDS-like style, which will evolve as connectivity derivatives naming conventions are codified.

The following code will run the *FSuB-Extractor* on data produced by code in this manuscript. Here, we use *FSuB-Extractor* to identify the sub-bundle of the arcuate fasciculus that connects to mOTS-words, a region that responds more to words compared to other categories of stimuli (defined in Section 2.7.4).

**Code Block 6: Example FSuB-Extractor command.**

*#!/bin/bash -l*
*## Define important paths and parameters*
bids="/path/to/nsd_bids/" *# Replace with your BIDS directory*
subject="sub-01" *# Subject name as found in FreeSurfer subjects directory*
freesurfer_dir=${bids}/derivatives/freesurfer/ *# FreeSurfer outputs*
qsirecon_dir=${bids}/derivatives/qsirecon/ *# DWI post-processing outputs*
tract="${qsirecon_dir}/${subject}/dwi/${subject}_space-T1w_desc-preproc/clean_bundles/${subject}_space-T1w_desc-preproc_dwi_space-RASMM_model-probCSD_algo-AFQ_desc-ARCL_tractography.trk"*# PyAFQ Left Arcuate Fasciculus, in this example*
tract_name="LeftArcuate" *# Descriptive tract name for file output names*
drawn_fROIs_dir=${bids}/derivatives/drawn_fROIs/
roi1="${drawn_fROIs_dir}/${subject}/${subject}_hemi-L_space-fsnative_contrast-charactersGTother_desc-mOTSwords_roi.func.gii"
*# A binary fROI on the FreeSurfer surface*
roi1_name="mOTS-words" *# A descriptive ROI name for file output names*
hemi="lh" *# FreeSurfer hemi name corresponding to the ROI*
out_dir=${bids}/derivatives/fsub_extractor/
xform_file="/path/to/sub-01_from-FS_to-T1wACPC_mode-image_xfm.txt" *# Transformation matrix from Freesurfer to DWI data (from script in supplementary materials S2)*

*### Run the FSuB-Extractor*
extractor \
  --subject $subject \
  --tract $tract \
  --tract-name $tract_name \
  --roi1 $roi1 \
  --roi1-name $roi1_name \
  --hemi $hemi \
  --out-dir $out_dir \
  --fs-dir $freesurfer_dir \
  --fs2dwi $xform_file

### 3.3 Validation

To validate the *FSuB-Extractor,* we reproduced the analysis pipeline from a previous study (Kubota et al., 2023), that used the FSuB approach with a custom MATLAB-based implementation that was originally presented in Grotheer et al. (2019). The study identified word and face-selective fROIs in individual children (*n* = 27) and adult (*n* = 28) participants and identified connections of each of these regions. To validate the software package, we used the same bundles, functional ROIs, and streamline-to-ROI association parameters (radial search with a search distance of 3 mm) that were defined in the original paper, and then used *FSuB-Extractor* to identify the connections of each of the functional ROIs.

**Figure 5:**
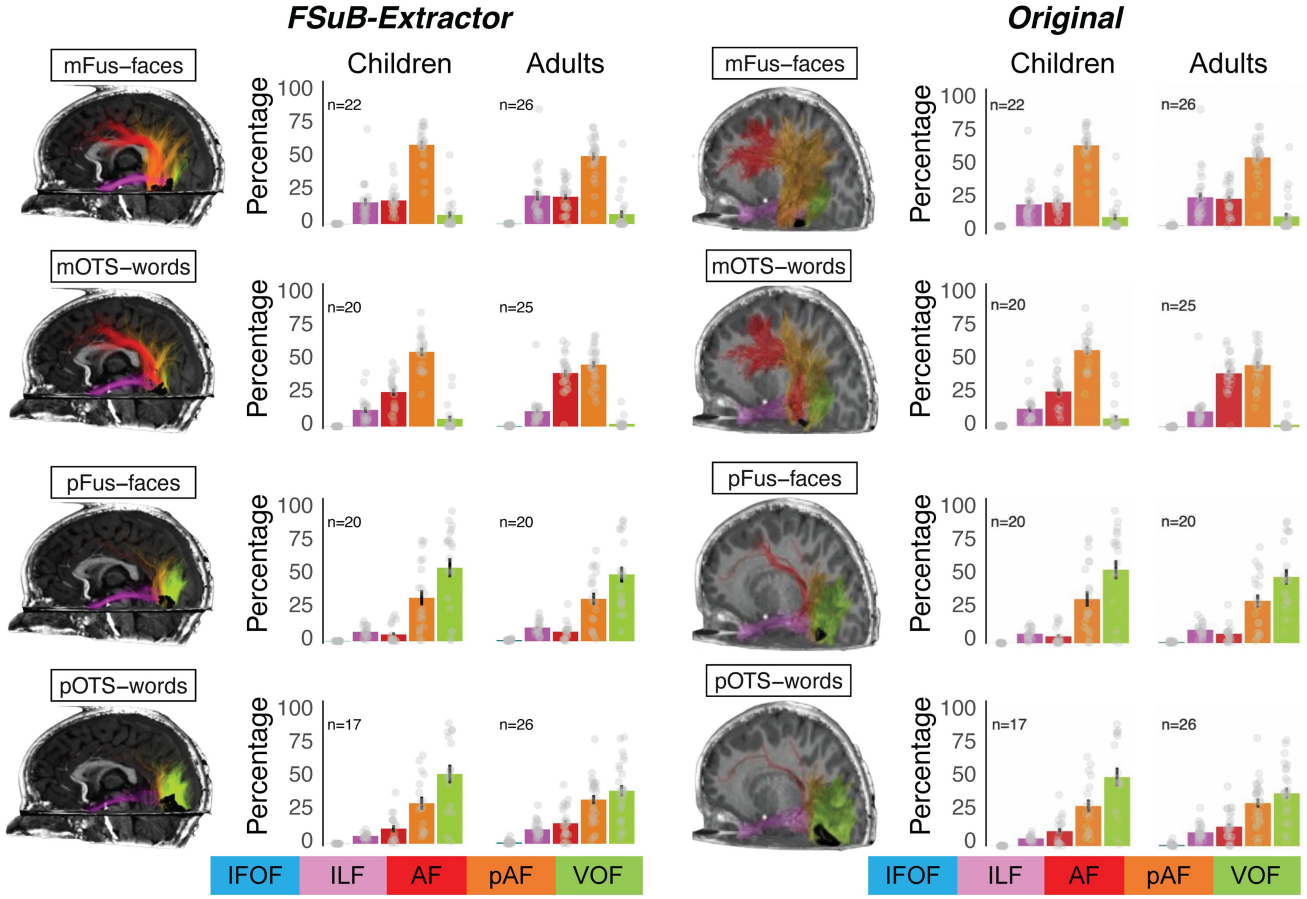
Validation of FSuB-Extractor. The FSuB-Extractor was used to reproduce findings from Kubota et al. (2023), looking at connectivity profiles of functionally defined sub-bundles in the human ventral visual stream among children and adults. The left half shows the results of the FSub-Extractor automated pipeline. The right half shows the original published data from Kubota et al. (2023). The brains depict FSuBs in a representative 6-year-old child participant for four functional fROIs (face selective: mFus-faces and pFus-faces; Word-selective: mOTS-words and pOTS-words). Acronyms: AF, Arcuate fasciculus; pAF, Posterior arcuate fasciculus; IFOF, Inferior fronto-occipital fasciculus; ILF, Inferior longitudinal fasciculus; VOF, ventral occipital fasciculus; mFus-faces, Mid-fusiform face-selective region; mOTS-words, mid-occipitotemporal sulcus word-selective region; pFus-faces, posterior fusiform face-selective region; pOTS-words, posterior occipitotemporal sulcus word-selective region.

We used *FSuB-Extractor* to identify the connections of each functional ROI and then defined the connections of each functional ROI as a “connectivity profile” or the percentage of streamlines associated with five bundles (the left arcuate, posterior arcuate, ventral occipital, inferior longitudinal, and inferior fronto-occipital fasciuli), as was done in the original paper. The original paper tested whether white matter connections of high-level visual areas were organized by stimulus-selective category or anatomical cytoarchitecture. In high-level visual cortex, there is not a one-to-one mapping between cytoarchitecture and category-selectivity (Weiner et al., 2017). mFus-faces and pFus-faces are both selective for faces, but located in different cytoarchitectonic areas (fusiform gyrus 4 (FG4) and fusiform gyrus 2 (FG2) respectively). Similarly, mOTS-words and pOTS-words are both selective for words and are located in different cytoarchitectonic areas (FG4 and FG2 respectively). Cytoarchitectonic area FG4 contains both a face- and a word-selective region (mFus-faces and mOTS-words), and cytoarchitectonic area FG2 contains both a face- and a word-selective region (pFus-faces and pOTS-words). In the original paper, it was found that white matter connections are more similar for regions located in the same cytoarchitectonic area, compared to regions with the same category selectivity. The connectivity profiles generated with *FSuB-Extractor* (Figure 5, left) reproduce these results (Figure 5, right). To quantify the similarity between connectivity profiles, we then calculated the correlation between the original connectivity profile and that generated using *FSuB-Extractor* for each fROI and each participant. We found that connectivity profiles were highly correlated using the two methods (mean correlation *r* = 0.99, standard deviation = 0.003). These results suggest that FSuB-Extractor is able to reproduce previous findings using the FSuB approach, and provides improvements as the code is open source, flexible, and easy to use.

## 4 Discussion

The present article suggests best practices for collecting and processing neuroimaging data for FSuB extraction, as well as provides a walkthrough of how to use a dedicated FSuB extraction software toolbox. Our toolbox is flexible, accepting multiple file types for gray matter fROIs and white matter tractograms. By not presuming any explicit organization for inputs, the user is not restricted to certain processing tools and can feasibly use their own data or publicly available preprocessed data. Over the past two decades, fMRI studies have identified regions in the cortex specialized for various *cognitive* functions (Kanwisher, 2010). Individual fascicles of the brain, on the other hand, are large and likely span multiple functional networks. The FSuB method will enable researchers to gain increased spatial precision in identifying the white matter involved in particular functional tasks (for a review see Grotheer et al., 2022). We hope our guide and software will facilitate the adoption of this approach, leading to new advances in precision neuroscience.

Aside from the inherent limitations of tractography (Bastiani et al., 2012; Thomas et al., 2014; Reveley et al., 2015; Maier-Hein et al., 2017) and fMRI (Logothetis, 2008), the practice of identifying FSuBs requires additional consideration because it relies on good registration between functional and diffusion data, sufficiently dense tractograms, and spatial proximity between functional regions and streamline endpoints. The suggestions in the present article will help optimize the approach. However, it is still essential that users have checks for quality assurance at each stage in their pipeline. In addition, users must take caution in their interpretation of results. For example, it is unlikely that very small FSuBs (e.g., a single streamline) should be interpreted as meaningful. Currently, the FSuB approach and the code in the present guide are best suited to cases where participants have high-quality diffusion and task-based functional MRI and a good surface reconstruction using *FreeSurfer*. This means that the software is suitable to use in typical, developmental, and clinical cohorts. However, in cases of lesions or atypical tissue, automatic segmentation methods may fail, which can hinder the validity of results. Due to the current state of the art of surface-based approaches in fMRI and DWI, the *FSuB-Extractor* is primarily limited to cortical applications. We hope to provide support for cerebellar and subcortical regions in the future. As the BIDS standards for derivatives change, we also plan to adapt our output naming conventions accordingly. We encourage any interested practitioners to contribute bug reports, feature requests, and code to help the *FSuB-Extractor* become more robust.

We note that our suggestions for handling data are summarized from prior empirical work that rigorously examined the impact of analysis parameters on processing outcomes (Glasser et al., 2013; Daducci et al., 2014; Canales-Rodríguez et al., 2019; Esteban et al., 2019; Yeh et al., 2019; Grisot et al., 2021; Maffei et al., 2022). Future work should examine the specific impacts of MRI acquisition and processing choices on FSuB outcomes. We hope that high-throughput analyses enabled by the FSuB-Extractor, coupled with future large datasets with sufficiently high-quality multimodal data and specific functional localization, will enable this kind of comprehensive parameter analysis.

Throughout the present article, fMRI has been the method to probe function in the brain. While fMRI is a ubiquitous tool in cognitive and perceptual neuroscience, other tools, such as positron emission tomography (PET), magnetoencephalography (MEG), and electroencephalography (EEG) are also commonly used. Although it has not been used for a FSuB purposes yet, an fROI derived from PET should work as long as the PET image can be successfully coregistered to a T1w image. MEG and EEG would not be recommended, as an fROI is hard to anatomically define due to ambiguous source localization.

An interesting potential application of the FSuB approach is in clinical practice. Functional neuroanatomy is an important consideration in surgical planning (that is, to target or preserve specific connections) and deep brain stimulation (modulating a behavior by stimulating a relevant bundle) (Essayed et al., 2017). However, functional MRI is rarely collected in pre-surgical patients, and DWI protocols often fall below the standards suggested in the present manuscript, which limits tractography’s utility (Essayed et al., 2017). Future work should evaluate the effectiveness of the FSuB approach on routine clinical data, with the ultimate goal of increasing the precision of surgical or stimulation targets or informing standards of clinical MRI collection.

One may also want to use the FSuB approach to identify the functional sub-bundles longitudinally. Importantly, FSuBs can be affected by both changes in a fROI location and changes in the white matter architecture. Therefore, it may be difficult to ascribe precise causes to observed changes in a FSuB over time. One remedy is to align longitudinal data to a within-subject template (Reuter et al., 2012). Using the functional data alone, it is possible to see whether the fROI is changing in size or location across timepoints. If the fROI is changing over time, it may result in differences in the underlying FSuB (and such developmental changes may be of interest by themselves without considering white matter). In order to identify changes in the white matter alone, it may be useful to hold the fROI constant, using a fROI from a single time point and combining it with diffusion data across sessions. For example, using a fROI from the final acquisition it is possible to “look back in time” and see how the white matter connections of this given region change across the study.

We clarify that our software is not the first or only way to combine DWI and fMRI images. Other approaches, such as track-weighted functional connectivity (Calamante et al., 2013) and the *Functionnectome* (Nozais et al., 2021, 2023), integrate fMRI derivatives with the underlying white matter architecture to draw valuable statistical inferences about functional neuroanatomy. Since these tools are primarily used for voxel- or region-wise between-subject inferences, these approaches necessitate data being in a common space or using a single normative set of white matter anatomical priors for inter-subject validity. What makes the FSuB approach unique in this respect is that each individual’s output is informed by participant-specific functional and anatomical patterns. The FSuB approach is particularly well suited for investigating functionally-targeted white matter micro- and macrostructural properties at the bundle level, while the aforementioned alternatives could be more appropriate for voxel-by-voxel inferences.

A topic that has been garnering increasing attention in the field of functional neuroanatomy is the white matter BOLD signal (for reviews, see Gawryluk et al., 2014; Gore et al., 2019). Despite this signal often being considered noise and regressed out during fMRI modeling, studies have suggested that the white matter BOLD signal is similarly time-locked to stimulus within task-relevant white matter pathways (Wu et al., 2017; Ding et al., 2018). Additionally, similar to DWI signal, white matter BOLD correlation patterns are anisotropic, in that a voxel’s BOLD time series will correlate more with an adjacent voxel in the same white matter pathway compare to an adjacent voxel in a different pathway (Ding et al., 2013). This pattern has been used to create BOLD directionality tensors (Ding et al., 2013), analogous to the diffusion tensor, and has even been extended to more complex models (Schilling et al., 2019b), analogous to how fiber orientation distributions are derived. These anisotropic representations of white matter BOLD have been used to recreate white matter pathways, with modest validity compared to conventional DWI tractography (Schilling et al., 2019b). Further methodological exploration into the white matter BOLD signal is warranted to characterize functional and anatomical specificity, and widespread adoption of white matter BOLD as a tool may be predicated on technological advances to effectively resolve the noisy signal. However, this approach has the potential to directly define FSuBs in white matter using fMRI, obviating the assumption that white matter pathways connecting to fROIs on the cortical surface must support the given task.

## 5 Conclusion

A better understanding of functional neuroanatomy can shed insights into large-scale neural networks and fine-grained cortical specialization that collectively orchestrate cognitive and perceptual processes in the brain. Multimodal neuroimaging can be used to resolve white matter at the level of functional sub-components, improving the anatomical and conceptual precision of brain structure–function-behavior studies. Researchers should be aware of methodological choices that impact the feasibility of this approach. We hope our guide, tutorial, and software will facilitate adoption of FSuB analyses.

## Data availability statement

The Natural Scenes Dataset (Allen et al., 2022) may be downloaded following directions at https://naturalscenesdataset.org/. Code displayed in this guide and a subset of data to run the analyses are available at https://osf.io/zf5q7/. Source code and installation instructions for the *FSuB-Extractor* may be found at https://github.com/smeisler/fsub_extractor.

## Ethics statement

The studies involving humans were approved by University of Minnesota Institutional Review Board. The studies were conducted in accordance with the local legislation and institutional requirements. The participants provided their written informed consent to participate in this study.

## Author contributions

SM: Conceptualization, Data curation, Formal analysis, Funding acquisition, Methodology, Software, Validation, Visualization, Writing – original draft, Writing – review & editing. EK: Conceptualization, Data curation, Formal analysis, Funding acquisition, Methodology, Software, Validation, Visualization, Writing – original draft, Writing – review & editing. MG: Conceptualization, Methodology, Supervision, Writing – review & editing. JG: Supervision, Writing – review & editing. KG-S: Conceptualization, Methodology, Supervision, Writing – review & editing.

## Footnotes

^1^ https://github.com/PennLINC/qsiprep

^3^ https://github.com/nipreps/fmriprep

^4^ https://www.nipreps.org/smriprep/

^5^ https://qsiprep.readthedocs.io/en/latest/reconstruction.html

^6^ https://www.humanconnectome.org/software/connectome-workbench

^7^ https://github.com/smeisler/fsub_extractor

^8^ https://github.com/smeisler/fsub_extractor/wiki

^9^ https://github.com/smeisler/fsub_extractor/issues
