# Supplementary material for: A practical guide for combining functional regions of interest and white matter bundles
Source: Front Neurosci. 2024 Aug 16;18:1385847. doi: 10.3389/fnins.2024.1385847 (PMC11363198; doi:10.3389/fnins.2024.1385847)
Supplement: Supplementary file 1 [file Data_Sheet_1.pdf]

Supplementary Material

For "**A practical guide for combining functional regions of interest and white matter bundles**" (by Meisler, Kubota, et al.)

S1. Singularity/Apptainer to Docker Code Conversion Example

Here, we demonstrate converting an example BIDS application container command between Singularity/Apptainer and Docker syntaxes. These two commands will run synonymous processes.

```
### First, Singularity/Apptainer
singularity run --containall -e \
    -B ${bids}:/bids \ # Bind BIDS directory to container, mounting as /bids
    -B ${workdir}:/work \ # Bind work directory to container, mounting as /work
    -B ${fs_license}:/license.txt \ Bind license to container, mounting as /license.txt
    /path/to/fmriprep_23.2.0a2.img \ # Container built with `singularity build` command
    /bids /bids/derivatives participants \ # Begin fMRIPrep arguments here
    -w /work --fs-license-file /license.txt \
    ${other_arguments_can_go_here}

### Now, Docker
docker run -ti --rm \
    -v ${bids}:/bids \ # Bind BIDS directory to container, mounting as /bids
    -v ${workdir}:/work \ # Bind work directory to container, mounting as /work
    -v ${fs_license}:/license.txt \ Bind license to container, mounting as /license.txt
    nipreps/fmriprep:23.2.0a2 # Name as found in corresponding DockerHub web page
    /bids /bids/derivatives participants \ # Begin fMRIPrep arguments here
    -w /work --fs-license-file /license.txt \
    ${other_arguments_can_go_here}
```

S2. Register *FreeSurfer* and *QSIPrep* Outputs

OSF path to file: `nsd_bids/code/qsiprep/fs_qsiprep_registration.sh`

```
#!/bin/bash -l
## Define important paths
bids="/path/to/nsd_bids/" # Or replace with your own BIDS dataset
workdir="/path/to/scratch/space/" # e.g., /tmp
qsiprep_IMG="/path/to/qsiprep_container.img" # Software container
subject="sub-01" # Or replace with your own subject ID
mkdir -p "${workdir}/${subject}"
export SUBJECTS_DIR="${bids}/derivatives/freesurfer/" # Where FS outputs are

## We can use the software in the QSIPrep container to run the commands below
run_qsiprep_cmd="singularity exec --containall -e -B ${bids},${workdir} ${qsiprep_IMG}" # Alias
for easy invocation of QSIPrep container

# Convert from FreeSurfer .mgz file format to NIFTI
```

```
${run_qsiprep_cmd} mrconvert -strides -1,-2,3 \
    ${SUBJECTS_DIR}/${subject}/mri/brain.mgz ${workdir}/${subject}/fs_brain.nii

# Register FreeSurfer brain to QSIPrep T1w
${run_qsiprep_cmd} antsRegistration --collapse-output-transforms 1 \
    --dimensionality 3 --float 0 \
    --initial-moving-transform [ ${bids}/derivatives/qsiprep/${subject}/anat/${subject}_desc-
preproc_T1w.nii, ${workdir}/${subject}/fs_brain.nii, 1 ] \
    --initialize-transforms-per-stage 0 --interpolation BSpline \
    --output [ ${workdir}/${subject}/transform, ${workdir}/${subject}/transform_Warped.nii.gz ]
\
    --transform Rigid[ 0.1 ] \
    --metric Mattes[ ${bids}/derivatives/qsiprep/${subject}/anat/${subject}_desc-
preproc_T1w.nii, ${workdir}/${subject}/fs_brain.nii, 1, 32, Random, 0.25 ] \
    --convergence [ 1000x500x250x100, 1e-06, 10 ] \
    --smoothing-sigmas 3.0x2.0x1.0x0.0mm --shrink-factors 8x4x2x1 \
    --use-histogram-matching 0 \
    --masks [ ${bids}/derivatives/qsiprep/${subject}/anat/${subject}_desc-brain_mask.nii.gz,
NULL ] \
    --winsorize-image-intensities [ 0.002, 0.998 ] \
    --write-composite-transform 0

# Convert ANTs .mat transform to .txt, and rename it
${run_qsiprep_cmd} ConvertTransformFile 3 \
    ${workdir}/${subject}/transform0GenericAffine.mat \
    ${workdir}/${subject}/${subject}_from-FS_to-T1wACPC_mode-image_xfm.txt

# Convert ANTs transform to MRTrix compatible transform
${run_qsiprep_cmd} transformconvert \
    ${workdir}/${subject}/${subject}_from-FS_to-T1wACPC_mode-image_xfm.txt \
    itk_import \
${bids}/derivatives/qsirecon/${subject}/anat/${subject}_from-FS_to-T1wACPC_mode-image_xfm.txt
```

S3. Surface-Based Analysis First-Level GLM

OSF path to file: `nsd_bids/code/l1_gifti/l1_gifti.ipynb`

```
import os.path as op
from os import makedirs
import numpy as np
from scipy import stats
import nibabel as nib
from nilearn.surface import load_surf_data
from nilearn.glm.first_level import (
    make_first_level_design_matrix,
    first_level_from_bids,
)
from nilearn.glm.first_level.first_level import run_glm
from nilearn.glm.contrasts import compute_contrast

### Helper function for saving GIFTI statmaps
def save_statmap_to_gifti(data, outname):
    """Save a statmap to a gifti file.
    data: nilearn contrast model output, e.g., contrast.effect_size()
```

```python
    outname: output file name
    """
    gii_to_save = nib.gifti.gifti.GiftiImage()
    gii_to_save.add_gifti_data_array(
        nib.gifti.gifti.GiftiDataArray(data=data, datatype="NIFTI_TYPE_FLOAT32")
    )
    nib.save(gii_to_save, outname)

### Define parameters here
bids = "/path/to/nsd_bids/"  # Path to BIDS root
fmriprep_dir = op.join(
    "derivatives", "fmriprep"
)  # BIDS-relative path to fMRIPrep
subject = "sub-01"  # Subject name
task = "floc"  # Task name
space = "fsnative"  # BOLD projected on subject's freesurfer surface
hemis = ["L", "R"]  # L for left, R for right
use_smoothed = False
run_nums = ["1", "2", "3", "4", "5", "6"] # Runs to process
slice_time_ref = (
    0  # From the fMRIPrep command, align slice time correction to start of TR
)

### Define output directory
outdir = op.join(bids, "derivatives", "l1_gifti", subject)
if not op.exists(outdir):
    makedirs(outdir)

### Loop across hemispheres
for hemi in hemis:
    print("Processing hemi", hemi)

    ### Final output dictionary for GLM contrast results (to be combined across runslater)
    contrast_objs = {}

    ### Loop over runs
    for run_num in run_nums:
        print("Processing run", run_num)

        ### Load GIFTI data and z-score it
        run = (
            "_run-" + run_num
        )  # Run string in filename (define as empty string "" if no run label)
        func_name = (
            f"{subject}_task-{task}{run}_hemi-{hemi}_space-{space}_bold.func.gii"
        )
        # If you smoothed data beforehand, make sure to point this to your smoothed file name!
        if use_smoothed:
            func_name = func_name.replace("_bold", "_desc-smoothed_bold")
        gii_path = op.join(bids, fmriprep_dir, subject, "func", func_name)
        gii_data = load_surf_data(gii_path)
        gii_data_std = stats.zscore(gii_data, axis=1)

        ### Get shape of data
```

```python
n_vertices = np.shape(gii_data)[0]
n_scans = np.shape(gii_data)[1]

### Use the volumetric data just to get the events and confounds file
img_filters = [("desc", "preproc")]
# If multiple runs are present, then add the run number to filter to specify
if len(run) > 0:
    img_filters.append(("run", run_num))
l1 = first_level_from_bids(
    bids,
    task,
    space_label="T1w",
    sub_labels=[subject[4:]],
    slice_time_ref=slice_time_ref,
    hrf_model="spm",
    drift_model=None,  # Do not high_pass since we use fMRIPrep's cosine regressors
    drift_order=0,  # Do not high_pass since we use fMRIPrep's cosine regressors
    high_pass=None,  # Do not high_pass since we use fMRIPrep's cosine regressors
    img_filters=img_filters,
    derivatives_folder=fmriprep_dir,
)

### Extract information from the prepared model
t_r = l1[0][0].t_r
events = l1[2][0][0]  # Dataframe of events information
confounds = l1[3][0][0]  # Dataframe of confounds

### From the confounds file, extract only those of interest
# Start with the motion and acompcor regressors
motion_keys = [
    "framewise_displacement",
    "rot_x",
    "rot_y",
    "rot_z",
    "trans_x",
    "trans_y",
    "trans_z",
]
# Get ACompCor components (all to explain 50% variance)
a_compcor_keys = [key for key in confounds.keys() if "a_comp_cor" in key]

# Now add non-steady-state volumes
non_steady_state_keys = [key for key in confounds.keys() if "non_steady" in key]

# Add cosine regressors which act to high-pass filter data at 1/128 Hz
cosine_keys = [key for key in confounds.keys() if "cosine" in key]

# Pull out the confounds we want to keep
confound_keys_keep = (
    motion_keys + a_compcor_keys + cosine_keys + non_steady_state_keys
)
confounds_keep = confounds[confound_keys_keep]

# Set first value of FD column to the column mean
```

```python
    confounds_keep["framewise_displacement"][0] = np.nanmean(
        confounds_keep["framewise_displacement"]
    )

    ### Create the design matrix
    # Start by getting times of scans
    frame_times = t_r * (np.arange(n_scans) + slice_time_ref)
    # Now use Nilearn to create the design matrix from the events files
    design_matrix = make_first_level_design_matrix(
        frame_times,
        events=events,
        hrf_model="spm",  # convolve with SPM's canonical HRF function
        drift_model=None,  # we use fMRIPrep's cosine regressors
        add_regs=confounds_keep,
    )

    # z-score the design matrix to standardize it
    design_matrix_std = stats.zscore(design_matrix, axis=0)
    # add constant in to standardized design matrix since you cannot z-score a constant
    design_matrix_std["constant"] = np.ones(len(design_matrix_std)).astype(int)

    ### Run the GLM
    Y = np.transpose(gii_data_std)
    X = np.asarray(design_matrix)
    labels, estimates = run_glm(Y, X, n_jobs=-1)

    ### Define the contrasts
    contrast_matrix = np.eye(design_matrix.shape[1])
    basic_contrasts = dict(
        [
            (column, contrast_matrix[i])
            for i, column in enumerate(design_matrix.columns)
        ]
    )
    contrasts = {
        "facesGTother": (
            basic_contrasts["adult"] / 2
            + basic_contrasts["child"] / 2
            - basic_contrasts["body"] / 8
            - basic_contrasts["limb"] / 8
            - basic_contrasts["number"] / 8
            - basic_contrasts["word"] / 8
            - basic_contrasts["car"] / 8
            - basic_contrasts["instrument"] / 8
            - basic_contrasts["corridor"] / 8
            - basic_contrasts["house"] / 8
        ),
        "charactersGTother": (
            basic_contrasts["number"] / 2
            + basic_contrasts["word"] / 2
            - basic_contrasts["body"] / 8
            - basic_contrasts["limb"] / 8
            - basic_contrasts["adult"] / 8
            - basic_contrasts["child"] / 8
```

```
                - basic_contrasts["car"] / 8
                - basic_contrasts["instrument"] / 8
                - basic_contrasts["corridor"] / 8
                - basic_contrasts["house"] / 8
            ),
            "placesGTother": (
                basic_contrasts["corridor"] / 2
                + basic_contrasts["house"] / 2
                - basic_contrasts["body"] / 8
                - basic_contrasts["limb"] / 8
                - basic_contrasts["adult"] / 8
                - basic_contrasts["child"] / 8
                - basic_contrasts["car"] / 8
                - basic_contrasts["instrument"] / 8
                - basic_contrasts["number"] / 8
                - basic_contrasts["word"] / 8
            ),
            "bodiesGTother": (
                basic_contrasts["body"] / 2
                + basic_contrasts["limb"] / 2
                - basic_contrasts["corridor"] / 8
                - basic_contrasts["house"] / 8
                - basic_contrasts["adult"] / 8
                - basic_contrasts["child"] / 8
                - basic_contrasts["car"] / 8
                - basic_contrasts["instrument"] / 8
                - basic_contrasts["number"] / 8
                - basic_contrasts["word"] / 8
            ),
            "objectsGTother": (
                basic_contrasts["car"] / 2
                + basic_contrasts["instrument"] / 2
                - basic_contrasts["corridor"] / 8
                - basic_contrasts["house"] / 8
                - basic_contrasts["adult"] / 8
                - basic_contrasts["child"] / 8
                - basic_contrasts["body"] / 8
                - basic_contrasts["limb"] / 8
                - basic_contrasts["number"] / 8
                - basic_contrasts["word"] / 8
            ),
        }

        ### Compute the contrasts
        for index, (contrast_id, contrast_val) in enumerate(contrasts.items()):
            # Add a label to the output dictionary if not present
            if contrast_id not in contrast_objs:
                contrast_objs[contrast_id] = []

            # Define a name template for output statistical maps (stat-X is replaced later on)
            outname_base = f"{subject}{run}_hemi-{hemi}_space-{space}_contrast-
{contrast_id}_stat-X_statmap.func.gii"
            if use_smoothed:
                outname_base = outname_base.replace(
```

```
                "_statmap", "_desc-smoothed_statmap"
            )
        outname_base = op.join(outdir, outname_base)  # Place in output directory

        # compute contrast-related statistics
        contrast = compute_contrast(
            labels, estimates, contrast_val, contrast_type="t"
        )
        # add contrast to the output dictionary
        contrast_objs[contrast_id].append(contrast)

        # do the run-specific processing
        betas = contrast.effect_size()
        z_score = contrast.z_score()
        t_value = contrast.stat()
        p_value = contrast.p_value()
        variance = contrast.effect_variance()

        # Save the value maps as GIFTIs
        # Effect size
        outname = outname_base.replace("stat-X", "stat-effect")
        save_statmap_to_gifti(betas, outname)

        # z-score
        outname = outname_base.replace("stat-X", "stat-z")
        save_statmap_to_gifti(z_score, outname)

        # t-value
        outname = outname_base.replace("stat-X", "stat-t")
        save_statmap_to_gifti(t_value, outname)

        # p-value
        outname = outname_base.replace("stat-X", "stat-p")
        save_statmap_to_gifti(p_value, outname)

        # variance
        outname = outname_base.replace("stat-X", "stat-variance")
        save_statmap_to_gifti(variance, outname)

### Now produce the session-wide statistical maps, averaging across all runs
if len(run_nums) > 1: # Only do if multiple runs are present
    print("Producing Session-Wide Statistical Maps")
    # Loop across contrast IDs
    for index, (contrast_id, contrast_val) in enumerate(contrasts.items()):
        # Add run-wide contrast objects together
        contrast_concat = contrast_objs[contrast_id][0]
        for i in range(1, len(contrast_objs[contrast_id])):
            contrast_concat = contrast_concat.__add__(contrast_objs[contrast_id][i])

        # Calculate the statistical maps
        betas = contrast_concat.effect_size()
        z_score = contrast_concat.z_score()
        t_value = contrast_concat.stat()
        p_value = contrast_concat.p_value()
```

```
            variance = contrast_concat.effect_variance()

            # Define output name template
            outname_base = f"{subject}_hemi-{hemi}_space-{space}_contrast-{contrast_id}_stat-
X_statmap.func.gii"
            if use_smoothed:
                outname_base = outname_base.replace("_statmap", "_desc-smoothed_statmap")
            outname_base = op.join(outdir, outname_base)

            # Save the value maps as GIFTIs
            # Effect size
            outname = outname_base.replace("stat-X", "stat-effect")
            save_statmap_to_gifti(betas, outname)

            # z-score
            outname = outname_base.replace("stat-X", "stat-z")
            save_statmap_to_gifti(z_score, outname)

            # t-value
            outname = outname_base.replace("stat-X", "stat-t")
            save_statmap_to_gifti(t_value, outname)

            # p-value
            outname = outname_base.replace("stat-X", "stat-p")
            save_statmap_to_gifti(p_value, outname)

            # variance
            outname = outname_base.replace("stat-X", "stat-variance")
            save_statmap_to_gifti(variance, outname)
```

S4. Volumetric Analysis

One can use *Fitlins* (https://github.com/poldracklab/fitlins) (Markiewicz et al., 2022) to run GLMs on the *fMRIPrep*-preprocessed volumetric data based on a model specification file and (https://bids-standard.github.io/stats-models/#) the BIDS events descriptor files in a subject's func folder. A model for the functional localizer task is shared below (as well as in the OSF repository). The model shared here will, within a given subject, calculate each categorically-selective functional contrast. Non-steady state volumes are censored, and confounds include framewise displacement, 6 head motion parameters, and anatomical CompCor component that explain 50% of signal variance in a combined white matter / cerebrospinal fluid mask(Behzadi et al., 2007) Cosine-basis functions are regressed, acting as a high-pass filter (1/128 seconds). Session-averaged statistical maps across all runs are also produced.

OSF path to file: `nsd_bids/models/model-floc_desc-6MP50ACompCor_smdl.json`

```
{
   "Name": "floc-model-6MP50ACompCor",
   "BIDSModelVersion": "1.0.0",
   "Description": "NSD contrasts; 6 Head Motion Parameters; FD; Non Steady-State;
50% Variance ACompCor Components",
   "Input": {
       "task": [
```

```
            "floc"
        ]
    },
    "Nodes": [
        {
            "Level": "run",
            "Name": "runFloc6MP50ACompCor",
            "GroupBy": [
                "subject",
                "session",
                "run"
            ],
            "Transformations": {
                "Transformer": "pybids-transforms-v1",
                "Instructions": [
                    {
                        "Name": "Factor",
                        "Input": [
                            "trial_type"
                        ]
                    },
                    {
                        "Name": "Convolve",
                        "Input": [
                            "trial_type.body",
                            "trial_type.limb",
                            "trial_type.number",
                            "trial_type.word",
                            "trial_type.adult",
                            "trial_type.child",
                            "trial_type.car",
                            "trial_type.instrument",
                            "trial_type.corridor",
                            "trial_type.house"
                        ],
                        "Model": "spm"
                    }
                ]
            },
            "Model": {
                "X": [
                    "trial_type.body",
                    "trial_type.limb",
                    "trial_type.number",
                    "trial_type.word",
                    "trial_type.adult",
                    "trial_type.child",
                    "trial_type.car",
                    "trial_type.instrument",
                    "trial_type.corridor",
                    "trial_type.house",
                    "non_steady_state*",
                    "framewise_displacement",
                    "trans_x",
```

```
                "trans_y",
                "trans_z",
                "rot_x",
                "rot_y",
                "rot_z",
                "a_comp_cor*",
                "cosine*",
                1
            ],
            "Type": "glm"
        },
        "Contrasts": [
            {
                "Name": "bodies_gt_other",
                "ConditionList": [
                    "trial_type.body",
                    "trial_type.limb",
                    "trial_type.number",
                    "trial_type.word",
                    "trial_type.adult",
                    "trial_type.child",
                    "trial_type.car",
                    "trial_type.instrument",
                    "trial_type.corridor",
                    "trial_type.house"
                ],
                "Weights": [
                    0.5,
                    0.5,
                    -0.125,
                    -0.125,
                    -0.125,
                    -0.125,
                    -0.125,
                    -0.125,
                    -0.125,
                    -0.125
                ],
                "Test": "t"
            },
            {
                "Name": "characters_gt_other",
                "ConditionList": [
                    "trial_type.body",
                    "trial_type.limb",
                    "trial_type.number",
                    "trial_type.word",
                    "trial_type.adult",
                    "trial_type.child",
                    "trial_type.car",
                    "trial_type.instrument",
                    "trial_type.corridor",
                    "trial_type.house"
                ],
```

```
                "Weights": [
                    -0.125,
                    -0.125,
                    0.5,
                    0.5,
                    -0.125,
                    -0.125,
                    -0.125,
                    -0.125,
                    -0.125,
                    -0.125
                ],
                "Test": "t"
            },
            {
                "Name": "faces_gt_other",
                "ConditionList": [
                    "trial_type.body",
                    "trial_type.limb",
                    "trial_type.number",
                    "trial_type.word",
                    "trial_type.adult",
                    "trial_type.child",
                    "trial_type.car",
                    "trial_type.instrument",
                    "trial_type.corridor",
                    "trial_type.house"
                ],
                "Weights": [
                    -0.125,
                    -0.125,
                    -0.125,
                    -0.125,
                    0.5,
                    0.5,
                    -0.125,
                    -0.125,
                    -0.125,
                    -0.125
                ],
                "Test": "t"
            },
            {
                "Name": "objects_gt_other",
                "ConditionList": [
                    "trial_type.body",
                    "trial_type.limb",
                    "trial_type.number",
                    "trial_type.word",
                    "trial_type.adult",
                    "trial_type.child",
                    "trial_type.car",
                    "trial_type.instrument",
                    "trial_type.corridor",
```

```
                "trial_type.house"
            ],
            "Weights": [
                -0.125,
                -0.125,
                -0.125,
                -0.125,
                -0.125,
                -0.125,
                0.5,
                0.5,
                -0.125,
                -0.125
            ],
            "Test": "t"
        },
        {
            "Name": "places_gt_other",
            "ConditionList": [
                "trial_type.body",
                "trial_type.limb",
                "trial_type.number",
                "trial_type.word",
                "trial_type.adult",
                "trial_type.child",
                "trial_type.car",
                "trial_type.instrument",
                "trial_type.corridor",
                "trial_type.house"
            ],
            "Weights": [
                -0.125,
                -0.125,
                -0.125,
                -0.125,
                -0.125,
                -0.125,
                -0.125,
                -0.125,
                0.5,
                0.5
            ],
            "Test": "t"
        }
    ]
},
{
    "Level": "session",
    "Name": "sessionFloc6MP50ACompCor",
    "GroupBy": [
        "session",
        "subject",
        "contrast"
    ],
```

```
            "Model": {
                "X": [
                    1
                ],
                "Type": "meta"
            },
            "DummyContrasts": {
                "Test": "t"
            }
        }
    ],
    "Edges": [
        {
            "Source": "runFloc6MP50ACompCor",
            "Destination": "sessionFloc6MP50ACompCor"
        }
    ]
}
```

We can run the model with the following code (based on *Fitlins* version 0.11.0):

```
#!/bin/bash -l
## Define important paths and parameters
bids="/path/to/nsd_bids/" # Or replace with your own BIDS dataset
workdir="/path/to/scratch/space/" # e.g., /tmp
fitlins_IMG="/path/to/fitlins_container.img" # Software container
subject="sub-01" # Or replace with your own subject ID
task="floc" # Task name of BOLD files
desc="6MP50ACompCor" # Model description
model_file={bids}/models/model-${task}_desc-${desc}_smdl.json
smoothing="4" # Smoothing kernel mm FWHM, can also set it at 0 for no smoothing
space="T1w" # Analyze the native space volumetric outputs
# Note: you can also process MNI or surface CIFTI files if available

# Run the FitLins command
singularity run --containall -e -B ${bids},${workdir} \ # Can also use Docker
    ${fitlins_IMG} ${bids} ${bids}/derivatives/fitlins participant \
    --participant-label ${subject} \ # Remove argument to process everyone
    -w ${workdir} \ # Working directory
    -m ${model_file} \ # Path to model specification
    -d ${bids}/derivatives/fmriprep/ # fMRIPrep outputs from earlier
    --space ${space} \ # Space of outputs
    -s ${smoothing} \ # Smoothing kernel
```

For an example of statistically thresholding an fROI, we can identify the voxels in the 90th percentile of *z*-scores for the character-selective contrast within the left fusiform gyrus, defined by the Automated Anatomical Labeling atlas (Tzourio-Mazoyer et al., 2002). Note this code uses the *Nilearn* (Abraham et al., 2014) and *ANTsPy* (Avants, Tustison & Song, 2009; Tustison et al., 2021) software packages.

```
import os.path as op
from os import makedirs
import numpy as np
from nilearn.image import load_img, new_img_like
from nilearn.datasets import fetch_atlas_aal
```

```python
from ants import apply_transforms, image_read, image_write

# Define parameters here
bids="/path/to/nsd_bids/" # Path to BIDS root
fmriprep_dir = op.join("derivatives","fmriprep") # BIDS-relative path to fMRIPrep
fitlins_dir = op.join("derivatives","fitlins") # BIDS-relative path to Fitlins
subject = "sub-01" # Subject name
node_name = "sessionFloc6MP50ACompCor" # Node name defined by Fitlins model
contrast_name = "charactersGtOther" # Contrast name defined by Fitlins model
percentile = 90 # Find top 10% of voxels in left IFG
out_dir = op.join(bids, 'derivatives', 'threshold_fROIs', subject)
if not op.exists(out_dir):
    makedirs(out_dir)
hemi = "L"
region = "Fusiform"
aal_regionname = f"Fusiform_{hemi}" # Region to extract
out_path = op.join(out_dir, f"{subject}_hemi-{hemi}_space-T1w_contrast-{contrast_name}_stat-
z_desc-{region}_desc-thresholded_roi.nii.gz") # Where to save the output image

### Load Z-stat image
z_img_path = op.join(bids,fitlins_dir,f"node-{node_name}",subject,
                          f"{subject}_contrast-{contrast_name}_stat-z_statmap.nii.gz")
z_img = load_img(z_img_path)
z_img_affine = z_img.affine # Affine matrix
z_img_values = z_img.get_fdata() # Array of data

### Register the AAL atlas to native space using ANTs
# Load the Z-stat img in a way that ANTs likes
z_img_ants = image_read(z_img_path)

# Download the AAL atlas
AAL = fetch_atlas_aal()
AAL_MNI_path = AAL.maps
AAL_MNI = image_read(AAL_MNI_path)

# Find the MNI-to-Native space transformation from fMRIPrep
MNI2Native_reg = op.join(bids,fmriprep_dir,subject,"anat",f"{subject}_from-
MNI152NLin2009cAsym_to-T1w_mode-image_xfm.h5")

# Run the registration and save out the image
AAL_native_path = op.join(bids,fmriprep_dir,subject,"anat",f"{subject}_desc-AAL_dseg.nii.gz")
# Where to save registered atlas
AAL_native = apply_transforms(z_img_ants, AAL_MNI, transformlist=[MNI2Native_reg],
interpolator="nearestNeighbor")
image_write(AAL_native, AAL_native_path)

### Get binary mask of the region
# Get index of AAL labels that contains "Fusiform_L"
region_index = AAL.labels.index(aal_regionname)
region_value = np.asarray(AAL.indices)[region_index].astype(int)
segmentation = load_img(AAL_native_path) # Load out native space segmentation
segmentation_values = segmentation.get_fdata()
# Make binary mask of all region values
region_mask_vals = np.full(np.shape(segmentation_values), False)
```

```
region_mask_vals[segmentation_values==region_value] = True
region_mask = new_img_like(z_img, region_mask_vals, affine=z_img_affine, copy_header=True)

### Find top 10% of values in region
z_img_values_masked = z_img_values[region_mask_vals]
percentile_thresh = np.percentile(z_img_values_masked, percentile) # The critical threshold
z_inds_gt_thresh_in_mask = (z_img_values>=percentile_thresh) * region_mask_vals
z_img_masked_thresholded = new_img_like(z_img, z_inds_gt_thresh_in_mask, affine=z_img_affine,
copy_header=True)
z_img_masked_thresholded.to_filename(out_path) # Save the image out
```

S5. Guide for Drawing fROIs

Here we provide a guide for hand drawing ROIs using *Freeview*. This functionality is only available in *Freeview* version >= 3 (corresponds to *FreeSurfer* version >= 7.0). The example fROI is mOTS-words which is named for its function (word-selective) and its anatomy (located in the mid-occipitotemporal sulcus region). We load the *z*-statistic map for the contrast of characters compared to other stimuli (*Figure S1, A*), threshold the map with a *z*-statistic > 3 (*Figure S1, B*), draw a path around the selected vertices that are above threshold and match our anatomical landmark of interest (*Figure S1, A*), close the path (*Figure S1, D and E*), and fill the ROI (*Figure S1, F*).

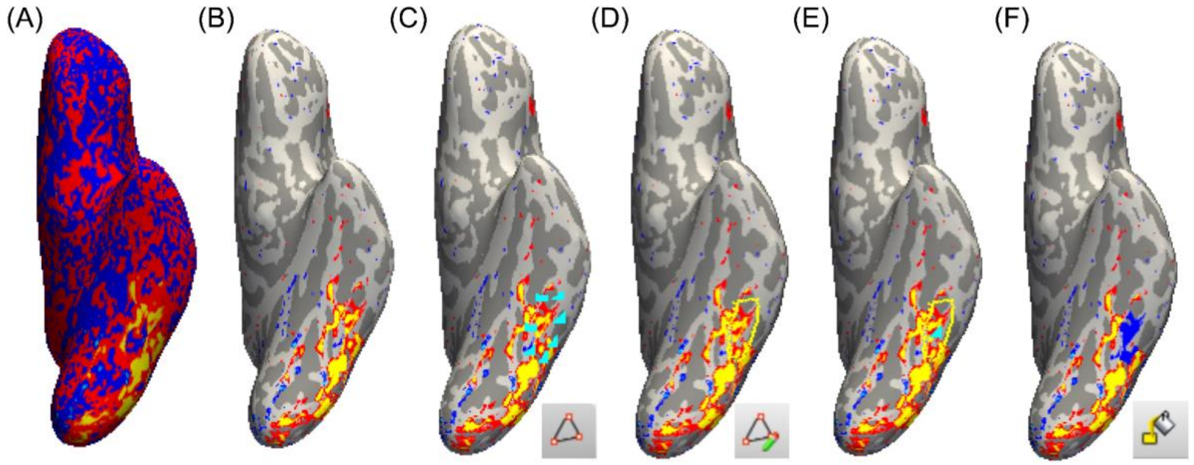

Figure S1. Tutorial for hand drawing ROIs. ***(A)*** *The first step is loading the statistical map. This map is the z-statistic contrast between response to characters compared to all other categories.* ***(B)*** *Next you can threshold the map, here we threshold with (z > 3).* ***(C)*** *Use the Path/Custom Fill tool to draw dots around your desired region of interest. Here we are defining mOTS-words so we choose vertices that are in the mid-occipital temporal sulcus region.* ***(D)*** *Use the Make Closed Path tool to close the path.* ***(E)*** *Put a dot in the center of your closed path.* ***(F)*** *Use the fill option to fill your closed path.*

S6. Detailed Pipeline Descriptions

The below boilerplate texts were automatically generated by *fMRIPrep* and *QSIPrep* with the express intention that users should copy and paste this text into their manuscripts unchanged. They are released under the CC0 license.

S6.1 Diffusion-Weighted Image Processing Pipeline

Preprocessing was performed using *QSIPrep* 0.19.1, which is based on *Nipype* 1.8.6 (Gorgolewski et al. (2011); Gorgolewski et al. (2018); RRID:SCR_002502).

S6.1.1 Anatomical data preprocessing

A total of 6 T1-weighted (T1w) images were found within the input BIDS dataset. All of them were corrected for intensity non-uniformity (INU) using `N4BiasFieldCorrection` (Tustison et al. 2010, *ANTs* 2.4.3). A T1w-reference map was computed after registration of 6 T1w images (after INU-correction) using `antsRegistration` [*ANTs* 2.4.3]. The anatomical reference image was reoriented into AC-PC alignment via a 6-DOF transform extracted from a full affine registration to the MNI152NLin2009cAsym template. A full nonlinear registration to the template from AC-PC space was estimated via symmetric nonlinear registration (SyN) using `antsRegistration`. Brain extraction was performed on the T1w image using `SynthStrip` (Hoopes et al. 2022) and automated segmentation was performed using `SynthSeg` (Billot, Greve, et al. 2023; Billot et al., 2023) from *FreeSurfer* version 7.3.1.

S6.1.2 Diffusion data preprocessing

Images were grouped into two phase encoding polarity groups. A total of 2 DWI series in the j+ phase-encoding direction distortion group were concatenated, with preprocessing operations performed on individual DWI series before concatenation. A total of 2 DWI series in the j- phase-encoding direction distortion group were concatenated, with preprocessing operations performed on individual DWI series before concatenation. Any images with a b-value less than 100 s/mm^2 were treated as a b=0 image. Denoising using `patch2self` (Fadnavis et al., 2020) was applied with settings based on developer recommendations. After `patch2self`, Gibbs unringing was performed using MRtrix3's `mrdegibbs` (Kellner et al. 2016). Following unringing, the mean intensity of the DWI series was adjusted so all the mean intensity of the b=0 images matched across each separate DWI scanning sequence. B1 field inhomogeneity was corrected using `dwibiascorrect` from *MRtrix3* with the N4 algorithm (Tustison et al. 2010) after corrected images were resampled. Both distortion groups were then merged into a single file, as required for the *FSL* workflows.

FSL (version 6.0.5.1:57b01774)'s `eddy` was used for head motion correction and Eddy current correction (Andersson and Sotiropoulos 2016). Eddy was configured with a q-space smoothing factor of 10, a total of 5 iterations, and 1000 voxels used to estimate hyperparameters. A linear first level model and a linear second level model were used to characterize Eddy current-related spatial distortion. q-space coordinates were forcefully assigned to shells. Field offset was attempted to be separated from subject movement. Shells were aligned post-eddy. Eddy's outlier replacement was run (Andersson et al. 2016). Data were grouped by slice, only including values from slices determined to contain at least 250 intracerebral voxels. Groups deviating by more than 4 standard deviations from the prediction had their data replaced with imputed values.

Data was collected with reversed phase-encode blips, resulting in pairs of images with distortions going in opposite directions. Here, multiple DWI series were acquired with opposite phase encoding directions. A b=0 image **and** the Fractional Anisotropy images from both phase encoding directions were used together in a multi-modal registration to estimate the susceptibility-induced off-resonance field. A T2-weighted image was included in the multimodal registration. An updated version of *DRBUDDI* (Irfanoglu et al., 2015), part of the *TORTOISE* (Irfanoglu et al., 2017) software package was used to estimate distortion. Signal intensity was adjusted in the final interpolated images using a method similar to LSR. Several confounding time-series were calculated based on the preprocessed DWI: framewise displacement (FD) using the implementation in *Nipype* (following the definitions by Power et al. 2014). The head-motion estimates calculated in the correction step were also placed within the corresponding confounds file. Slicewise cross correlation was also calculated. The DWI time-series were resampled to ACPC, generating a *preprocessed DWI run in ACPC space* with 1.25mm isotropic voxels.

Many internal operations of *QSIPrep* use *Nilearn* 0.10.2 (Abraham et al. 2014, RRID:SCR_001362) and *DIPY* (Garyfallidis et al. 2014). For more details of the pipeline, see the section corresponding to workflows in *QSIPrep's* documentation.

S6.1.3 Diffusion data postprocessing

Reconstruction was performed using *QSIprep* 0.19.1, which is based on *Nipype* 1.8.6 (Gorgolewski et al. (2011); Gorgolewski et al. (2018); RRID:SCR_002502).

QSIPrep-preprocessed T1w images and brain masks were used. A hybrid surface/volume segmentation was created [Smith 2020]. *FreeSurfer* outputs were registered to the *QSIPrep* outputs. Multi-tissue fiber response functions were estimated using the `dhollander` algorithm (Dhollander et al., 2019). FODs were estimated via constrained spherical deconvolution (CSD, Tournier et al., 2007, 2008) using an unsupervised multi-tissue method (Dhollander et al., 2016, 2019). Reconstruction was done using *MRtrix3* (Tournier et al., 2019). FODs were intensity-normalized using `mtnormalize` (Dhollander et al., 2021). The FODs and surface/volume segmentation were used for anatomically-constrained `iFOD2` tractography (Smith et al., 2012, 2020). 10 million streamlines with a length of 30-250 mm were created, with backtracking and cropping at the gray matter white matter interface enabled. These streamlines were then fed into *PyAFQ* to segment major white matter bundles (Kruper et al., 2021).

S6.2 Functional MRI Processing Pipeline

Results included in this manuscript come from preprocessing performed using *fMRIPrep* 23.2.0a2 (Esteban et al. (2019); Esteban et al. (2018); RRID:SCR_016216), which is based on *Nipype* 1.8.6 (K. Gorgolewski et al. (2011); K. J. Gorgolewski et al. (2018); RRID:SCR_002502).

S6.2.1 Preprocessing of B0 inhomogeneity mappings

A total of 3 fieldmaps were found available within the input BIDS structure for this particular subject. A B0 nonuniformity map (or fieldmap) was estimated from the phase-drift map(s) measure

with two consecutive GRE (gradient-recalled echo) acquisitions. The corresponding phase-map(s) were phase-unwrapped with `prelude` (*FSL*).

S6.2.2 Anatomical data preprocessing

A total of 6 T1-weighted (T1w) images were found within the input BIDS dataset. Each T1w image was corrected for intensity non-uniformity (INU) with `N4BiasFieldCorrection` (Tustison et al. 2010), distributed with *ANTs* 2.5.0 (Avants et al. 2008, RRID:SCR_004757). The T1w-reference was then skull-stripped with a *Nipype* implementation of the `antsBrainExtraction.sh` workflow (from *ANTs*), using *OASIS30ANTs* as target template. Brain tissue segmentation of cerebrospinal fluid (CSF), white-matter (WM) and gray-matter (GM) was performed on the brain-extracted T1w using `fast` (*FSL* (version unknown), RRID:SCR_002823, Zhang, Brady, and Smith 2001). An anatomical T1w-reference map was computed after registration of 6 T1w images (after INU-correction) using `mri_robust_template` (*FreeSurfer* 7.3.2, Reuter, Rosas, and Fischl 2010). An anatomical T2w-reference map was computed after registration of 3 T2w images (after INU-correction) using `mri_robust_template` (*FreeSurfer* 7.3.2, Reuter, Rosas, and Fischl 2010). Brain surfaces were reconstructed using `recon-all` (*FreeSurfer* 7.3.2, RRID:SCR_001847, Dale, Fischl, and Sereno 1999), and the brain mask estimated previously was refined with a custom variation of the method to reconcile *ANTs*-derived and *FreeSurfer*-derived segmentations of the cortical gray-matter of *Mindboggle* (RRID:SCR_002438, Klein et al. 2017). A T2-weighted image was used to improve pial surface refinement. Brain surfaces were reconstructed using recon-all (*FreeSurfer* 7.3.2, RRID:SCR_001847, Dale, Fischl, and Sereno 1999), and the brain mask estimated previously was refined with a custom variation of the method to reconcile *ANT*s-derived and *FreeSurfer*-derived segmentations of the cortical gray-matter of *Mindboggle* (RRID:SCR_002438, Klein et al. 2017). Volume-based spatial normalization to two standard spaces (MNI152NLin2009cAsym, MNI152NLin6Asym) was performed through nonlinear registration with `antsRegistration` (*ANTs* 2.5.0), using brain-extracted versions of both T1w reference and the T1w template. The following templates were were selected for spatial normalization and accessed with *TemplateFlow* (23.1.0, Ciric et al. 2022): ICBM 152 Nonlinear Asymmetrical template version 2009c [Fonov et al. (2009), RRID:SCR_008796; TemplateFlow ID: MNI152NLin2009cAsym], FSL's MNI ICBM 152 non-linear 6th Generation Asymmetric Average Brain Stereotaxic Registration Model [Evans et al. (2012), RRID:SCR_002823; TemplateFlow ID: MNI152NLin6Asym]. Grayordinate "dscalar" files containing 91k samples were resampled onto *fsLR* using the Connectome Workbench (Glasser et al. 2013).

S6.2.3 Functional data preprocessing

For each of the 6 BOLD runs found per subject (across all tasks and sessions), the following preprocessing was performed. First, a reference volume was generated, using a custom methodology of *fMRIPrep*, for use in head motion correction. Head-motion parameters with respect to the BOLD reference (transformation matrices, and six corresponding rotation and translation parameters) are estimated before any spatiotemporal filtering using `mcflirt` (*FSL*, Jenkinson et al. 2002). The estimated fieldmap was then aligned with rigid-registration to the target EPI (echo-planar imaging) reference run. The field coefficients were mapped on to the reference EPI using the transform. The BOLD reference was then co-registered to the T1w reference using

`bbregister` (*FreeSurfer*) which implements boundary-based registration (Greve and Fischl 2009). Co-registration was configured with six degrees of freedom. Several confounding time-series were calculated based on the preprocessed BOLD: framewise displacement (FD), DVARS and three region-wise global signals. FD was computed using two formulations following Power (absolute sum of relative motions, Power et al. (2014)) and Jenkinson (relative root mean square displacement between affines, Jenkinson et al. (2002)). FD and DVARS are calculated for each functional run, both using their implementations in *Nipype* (following the definitions by Power et al. 2014). The three global signals are extracted within the CSF, the WM, and the whole-brain masks. Additionally, a set of physiological regressors were extracted to allow for component-based noise correction (CompCor, Behzadi et al. 2007). Principal components are estimated after high-pass filtering the preprocessed BOLD time-series (using a discrete cosine filter with 128s cut-off) for the two CompCor variants: temporal (tCompCor) and anatomical (aCompCor). tCompCor components are then calculated from the top 2% variable voxels within the brain mask. For aCompCor, three probabilistic masks (CSF, WM and combined CSF+WM) are generated in anatomical space. The implementation differs from that of Behzadi et al. in that instead of eroding the masks by 2 pixels on BOLD space, a mask of pixels that likely contain a volume fraction of GM is subtracted from the aCompCor masks. This mask is obtained by dilating a GM mask extracted from the *FreeSurfer*'s aseg segmentation, and it ensures components are not extracted from voxels containing a minimal fraction of GM. Finally, these masks are resampled into BOLD space and binarized by thresholding at 0.99 (as in the original implementation). Components are also calculated separately within the WM and CSF masks. For each CompCor decomposition, the *k* components with the largest singular values are retained, such that the retained components' time series are sufficient to explain 50 percent of variance across the nuisance mask (CSF, WM, combined, or temporal). The remaining components are dropped from consideration. The head-motion estimates calculated in the correction step were also placed within the corresponding confounds file. The confound time series derived from head motion estimates and global signals were expanded with the inclusion of temporal derivatives and quadratic terms for each (Satterthwaite et al. 2013). Frames that exceeded a threshold of 0.5 mm FD or 1.5 standardized DVARS were annotated as motion outliers. Additional nuisance timeseries are calculated by means of principal components analysis of the signal found within a thin band (crown) of voxels around the edge of the brain, as proposed by (Patriat, Reynolds, and Birn 2017). The BOLD time-series were resampled onto the following surfaces (*FreeSurfer* reconstruction nomenclature): fsnative. The BOLD time-series were resampled onto the left/right-symmetric template "fsLR" using the Connectome Workbench (Glasser et al. 2013). A "goodvoxels" mask was applied during volume-to-surface sampling in fsLR space, excluding voxels whose time-series have a locally high coefficient of variation. *Grayordinates* files (Glasser et al. 2013) containing 91k samples were also generated with surface data transformed directly to fsLR space and subcortical data transformed to 2 mm resolution MNI152NLin6Asym space. All resamplings can be performed with a single interpolation step by composing all the pertinent transformations (i.e. head-motion transform matrices, susceptibility distortion correction when available, and co-registrations to anatomical and output spaces). Gridded (volumetric) resamplings were performed using *nitransforms*, configured with cubic B-spline interpolation. Non-gridded (surface) resamplings were performed using `mri_vol2surf` (*FreeSurfer*).

Many internal operations of *fMRIPrep* use *Nilearn* 0.10.2 (Abraham et al. 2014, RRID:SCR_001362), mostly within the functional processing workflow. For more details of the pipeline, see the section corresponding to workflows in *fMRIPrep's* documentation.

Supplemental References

Abraham, Alexandre, Fabian Pedregosa, Michael Eickenberg, Philippe Gervais, Andreas Mueller, Jean Kossaifi, Alexandre Gramfort, Bertrand Thirion, and Gael Varoquaux. 2014. "Machine Learning for Neuroimaging with Scikit-Learn." *Frontiers in Neuroinformatics* 8. https://doi.org/10.3389/fninf.2014.00014.

Andersson, Jesper LR, and Stamatios N Sotiropoulos. 2016. "An Integrated Approach to Correction for Off-Resonance Effects and Subject Movement in Diffusion Mr Imaging." *Neuroimage* 125. Elsevier: 1063–78.

Andersson, Jesper LR, Mark S Graham, Enikő Zsoldos, and Stamatios N Sotiropoulos. 2016. "Incorporating Outlier Detection and Replacement into a Non-Parametric Framework for Movement and Distortion Correction of Diffusion Mr Images." *Neuroimage* 141. Elsevier: 556–72.

Andersson, Jesper LR, Stefan Skare, and John Ashburner. 2003. "How to Correct Susceptibility Distortions in Spin-Echo Echo-Planar Images: Application to Diffusion Tensor Imaging." *Neuroimage* 20 (2). Elsevier: 870–88.

Avants, B. B., C. L. Epstein, M. Grossman, and J. C. Gee. 2008. "Symmetric Diffeomorphic Image Registration with Cross-Correlation: Evaluating Automated Labeling of Elderly and Neurodegenerative Brain." *Medical Image Analysis* 12 (1): 26–41. https://doi.org/10.1016/j.media.2007.06.004.

Avants, B.B., Tustison, N. & Song, G. (2009) Advanced normalization tools (ANTS). *Insight j*. 2 (365), 1–35.

Behzadi, Yashar, Khaled Restom, Joy Liau, and Thomas T. Liu. 2007. "A Component Based Noise Correction Method (CompCor) for BOLD and Perfusion Based fMRI." *NeuroImage* 37 (1): 90–101. https://doi.org/10.1016/j.neuroimage.2007.04.042.

Billot, Benjamin, Douglas N Greve, Oula Puonti, Axel Thielscher, Koen Van Leemput, Bruce Fischl, Adrian V Dalca, Juan Eugenio Iglesias, and others. 2023. "SynthSeg: Segmentation of Brain Mri Scans of Any Contrast and Resolution Without Retraining." *Medical Image Analysis* 86. Elsevier: 102789.

Billot, Benjamin, Colin Magdamo, You Cheng, Steven E Arnold, Sudeshna Das, and Juan Eugenio Iglesias. 2023. "Robust Machine Learning Segmentation for Large-Scale Analysis of Heterogeneous Clinical Brain Mri Datasets." *Proceedings of the National Academy of Sciences* 120 (9). National Acad Sciences: e2216399120.

Ciric, R., William H. Thompson, R. Lorenz, M. Goncalves, E. MacNicol, C. J. Markiewicz, Y. O. Halchenko, et al. 2022. "TemplateFlow: FAIR-Sharing of Multi-Scale, Multi-Species Brain Models." Nature Methods 19: 1568–71. https://doi.org/10.1038/s41592-022-01681-2.

Dale, Anders M., Bruce Fischl, and Martin I. Sereno. 1999. "Cortical Surface-Based Analysis: I. Segmentation and Surface Reconstruction." *NeuroImage* 9 (2): 179–94. https://doi.org/10.1006/nimg.1998.0395.

Dhollander, Thijs, David Raffelt, and Alan Connelly. "Unsupervised 3-tissue response function estimation from single-shell or multi-shell diffusion MR data without a co-registered T1 image." In *ISMRM workshop on breaking the barriers of diffusion MRI*, vol. 5, no. 5. 2016.

Dhollander, Thijs, Rami Tabbara, Jonas Rosnarho-Tornstrand, Jacques-Donald Tournier, David Raffelt, and Alan Connelly. "Multi-tissue log-domain intensity and inhomogeneity normalisation for quantitative apparent fibre density." In *Proc. ISMRM*, vol. 29, p. 2472. 2021.

Dhollander, Thijs, Remika Mito, David Raffelt, and Alan Connelly. "Improved white matter response function estimation for 3-tissue constrained spherical deconvolution." In *Proc. Intl. Soc. Mag. Reson. Med*, vol. 555, no. 10. 2019.

Esteban, Oscar, Ross Blair, Christopher J. Markiewicz, Shoshana L. Berleant, Craig Moodie, Feilong Ma, Ayse Ilkay Isik, et al. 2018. "fMRIPrep 23.2.1." Software. https://doi.org/10.5281/zenodo.852659.

Esteban, Oscar, Christopher Markiewicz, Ross W Blair, Craig Moodie, Ayse Ilkay Isik, Asier Erramuzpe Aliaga, James Kent, et al. 2019. "fMRIPrep: A Robust Preprocessing Pipeline for Functional MRI." *Nature Methods* 16: 111–16. https://doi.org/10.1038/s41592-018-0235-4.

Evans, AC, AL Janke, DL Collins, and S Baillet. 2012. "Brain Templates and Atlases." NeuroImage 62 (2): 911–22. https://doi.org/10.1016/j.neuroimage.2012.01.024.

Fadnavis, Shreyas, Joshua Batson, and Eleftherios Garyfallidis. "Patch2Self: Denoising Diffusion MRI with Self-Supervised Learning." *Advances in Neural Information Processing Systems* 33 (2020): 16293-16303.

Fonov, VS, AC Evans, RC McKinstry, CR Almli, and DL Collins. 2009. "Unbiased Nonlinear Average Age-Appropriate Brain Templates from Birth to Adulthood." *NeuroImage* 47, Supplement 1: S102. https://doi.org/10.1016/S1053-8119(09)70884-5.

Garyfallidis, Eleftherios, Matthew Brett, Bagrat Amirbekian, Ariel Rokem, Stefan Van Der Walt, Maxime Descoteaux, and Ian Nimmo-Smith. 2014. "Dipy, a Library for the Analysis of Diffusion Mri Data." *Frontiers in Neuroinformatics* 8. Frontiers: 8.

Glasser, Matthew F., Stamatios N. Sotiropoulos, J. Anthony Wilson, Timothy S. Coalson, Bruce Fischl, Jesper L. Andersson, Junqian Xu, et al. 2013. "The Minimal Preprocessing Pipelines for the Human Connectome Project." *NeuroImage*, Mapping the connectome, 80: 105–24. https://doi.org/10.1016/j.neuroimage.2013.04.127.

Gorgolewski, K., C. D. Burns, C. Madison, D. Clark, Y. O. Halchenko, M. L. Waskom, and S. Ghosh. 2011. “Nipype: A Flexible, Lightweight and Extensible Neuroimaging Data Processing Framework in Python.” *Frontiers in Neuroinformatics* 5: 13. https://doi.org/10.3389/fninf.2011.00013.

Gorgolewski, Krzysztof J., Oscar Esteban, Christopher J. Markiewicz, Erik Ziegler, David Gage Ellis, Michael Philipp Notter, Dorota Jarecka, et al. 2018. “Nipype.” Software. https://doi.org/10.5281/zenodo.596855.

Greve, Douglas N, and Bruce Fischl. 2009. “Accurate and Robust Brain Image Alignment Using Boundary-Based Registration.” *NeuroImage* 48 (1): 63–72. https://doi.org/10.1016/j.neuroimage.2009.06.060.

Hoopes, Andrew, Jocelyn S Mora, Adrian V Dalca, Bruce Fischl, and Malte Hoffmann. 2022. “SynthStrip: Skull-Stripping for Any Brain Image.” *NeuroImage* 260. Elsevier: 119474.

Irfanoglu, Mustafa Okan, Amritha Nayak, Jeffrey Jenkins, and Carlo Pierpaoli. "TORTOISE v3: Improvements and new features of the NIH diffusion MRI processing pipeline." In *Program and proceedings of the ISMRM 25th annual meeting and exhibition, Honolulu, HI, USA*. 2017.

Irfanoglu, Mustafa Okan, Pooja Modi, Amritha Nayak, Elizabeth B. Hutchinson, Joelle Sarlls, and Carlo Pierpaoli. "DR-BUDDI (Diffeomorphic Registration for Blip-Up blip-Down Diffusion Imaging) method for correcting echo planar imaging distortions." *Neuroimage* 106 (2015): 284-299.

Jenkinson, Mark, Peter Bannister, Michael Brady, and Stephen Smith. 2002. “Improved Optimization for the Robust and Accurate Linear Registration and Motion Correction of Brain Images.” *NeuroImage* 17 (2): 825–41. https://doi.org/10.1006/nimg.2002.1132.

Kellner, Elias, Bibek Dhital, Valerij G Kiselev, and Marco Reisert. 2016. “Gibbs-Ringing Artifact Removal Based on Local Subvoxel-Shifts.” *Magnetic Resonance in Medicine* 76 (5). Wiley Online Library: 1574–81.

Klein, Arno, Satrajit S. Ghosh, Forrest S. Bao, Joachim Giard, Yrjö Häme, Eliezer Stavsky, Noah Lee, et al. 2017. “Mindboggling Morphometry of Human Brains.” PLOS Computational Biology 13 (2): e1005350. https://doi.org/10.1371/journal.pcbi.1005350.

Kruper, John, Jason D. Yeatman, Adam Richie-Halford, David Bloom, Mareike Grotheer, Sendy Caffarra, Gregory Kiar et al. "Evaluating the reliability of human brain white matter tractometry." *Aperture neuro* 1, no. 1 (2021).

Markiewicz, C.J., De La Vega, A., Wagner, A., Halchenko, Y.O., Finc, K., Ciric, R., Goncalves, M., Nielson, D.M., Kent, J.D., Lee, J.A., Bansal, S., Poldrack, R.A. & Gorgolewski, K.J. (2022) *poldracklab/fitlins: 0.11.0*. doi:10.5281/zenodo.1306215.

Patriat, Rémi, Richard C. Reynolds, and Rasmus M. Birn. 2017. “An Improved Model of Motion-Related Signal Changes in fMRI.” *NeuroImage* 144, Part A (January): 74–82. https://doi.org/10.1016/j.neuroimage.2016.08.051.

Power, Jonathan D., Anish Mitra, Timothy O. Laumann, Abraham Z. Snyder, Bradley L. Schlaggar, and Steven E. Petersen. 2014. “Methods to Detect, Characterize, and Remove Motion Artifact in Resting State fMRI.” *NeuroImage* 84 (Supplement C): 320–41. https://doi.org/10.1016/j.neuroimage.2013.08.048.

Reuter, Martin, Herminia Diana Rosas, and Bruce Fischl. 2010. “Highly Accurate Inverse Consistent Registration: A Robust Approach.” *NeuroImage* 53 (4): 1181–96. https://doi.org/10.1016/j.neuroimage.2010.07.020.

Satterthwaite, Theodore D., Mark A. Elliott, Raphael T. Gerraty, Kosha Ruparel, James Loughead, Monica E. Calkins, Simon B. Eickhoff, et al. 2013. “An improved framework for confound regression and filtering for control of motion artifact in the preprocessing of resting-state functional connectivity data.” *NeuroImage* 64 (1): 240–56. https://doi.org/10.1016/j.neuroimage.2012.08.052.

Smith, Robert, Antonin Skoch, Claude J. Bajada, Svenja Caspers, and Alan Connelly. "Hybrid surface-volume segmentation for improved anatomically-constrained tractography." (2020).

Smith, Robert E., Jacques-Donald Tournier, Fernando Calamante, and Alan Connelly. "Anatomically-constrained tractography: improved diffusion MRI streamlines tractography through effective use of anatomical information." *Neuroimage* 62, no. 3 (2012): 1924-1938.

Tournier, J-Donald, Chun-Hung Yeh, Fernando Calamante, Kuan-Hung Cho, Alan Connelly, and Ching-Po Lin. "Resolving crossing fibres using constrained spherical deconvolution: validation using diffusion-weighted imaging phantom data." *Neuroimage* 42, no. 2 (2008): 617-625.

Tournier, J-Donald, Fernando Calamante, and Alan Connelly. "Robust determination of the fibre orientation distribution in diffusion MRI: non-negativity constrained super-resolved spherical deconvolution." *Neuroimage* 35, no. 4 (2007): 1459-1472.

Tournier, J. Donald, Fernando Calamante, and Alan Connelly. "Improved probabilistic streamlines tractography by 2nd order integration over fibre orientation distributions." In *Proceedings of the international society for magnetic resonance in medicine*, vol. 1670. New Jersey, USA: John Wiley & Sons, Inc, 2010.

Tournier, J-Donald, Robert Smith, David Raffelt, Rami Tabbara, Thijs Dhollander, Maximilian Pietsch, Daan Christiaens, Ben Jeurissen, Chun-Hung Yeh, and Alan Connelly. "MRtrix3: A fast, flexible and open software framework for medical image processing and visualisation." *Neuroimage* 202 (2019): 116137.

Tustison, N. J., B. B. Avants, P. A. Cook, Y. Zheng, A. Egan, P. A. Yushkevich, and J. C. Gee. 2010. “N4ITK: Improved N3 Bias Correction.” *IEEE Transactions on Medical Imaging* 29 (6): 1310–20. https://doi.org/10.1109/TMI.2010.2046908.

Tustison, N.J., Cook, P.A., Holbrook, A.J., Johnson, H.J., Muschelli, J., Devenyi, G.A., Duda, J.T., Das, S.R., Cullen, N.C. & Gillen, D.L. (2021) The ANTsX ecosystem for quantitative biological and medical imaging. *Scientific reports*. 11 (1), 1–13.

Tzourio-Mazoyer, N., Landeau, B., Papathanassiou, D., Crivello, F., Etard, O., Delcroix, N., Mazoyer, B. & Joliot, M. (2002) Automated Anatomical Labeling of Activations in SPM Using a Macroscopic Anatomical Parcellation of the MNI MRI Single-Subject Brain. *NeuroImage*. 15 (1), 273–289. doi:10.1006/nimg.2001.0978.

Zhang, Y., M. Brady, and S. Smith. 2001. “Segmentation of Brain MR Images Through a Hidden Markov Random Field Model and the Expectation-Maximization Algorithm.” *IEEE Transactions on Medical Imaging* 20 (1): 45–57. https://doi.org/10.1109/42.906424.
